# Biomimetic Mammary Gland Organoid-on-a-Chip for Producing Selected Human Milk Components

**DOI:** 10.34133/research.1356

**Published:** 2026-07-17

**Authors:** Xiang Lin, Yi Cheng, Junqi Zhao, Luoran Shang, Yuanjin Zhao

**Affiliations:** ^1^ Department of Rheumatology and Immunology, Nanjing Drum Tower Hospital, School of Biological Science and Medical Engineering, Southeast University, Nanjing 210096, China.; ^2^ Shanghai Xuhui Central Hospital, Zhongshan-Xuhui Hospital, and The Shanghai Key Laboratory of Medical Epigenetics, The International Co-laboratory of Medical Epigenetics and Metabolism (Ministry of Science and Technology), Institutes of Biomedical Sciences, Fudan University, Shanghai 200032, China.; ^3^ Wenzhou Institute, University of Chinese Academy of Sciences, Wenzhou, Zhejiang 325001, China.

## Abstract

Milk is an essential nutritional source for human health, yet developing efficient and sustainable indoor dairy production systems to produce milk-associated components remains a major challenge. Here, we propose a biomimetic mammary gland-on-a-chip system that recapitulates both the structural features and lactation mechanism of the mammary gland for producing milk-associated bioactive components. The chip is designed with a medium channel providing circulation, an intermediate porous hydrogel layer for substance diffusion, and highly specialized microchambers for culturing mammary epithelial cells. The mammary gland-on-a-chip enables tight-junction formation and active secretion of milk bioactive components such as lactotransferrin and triglycerides. More importantly, by expanding the microchambers and parallelizing the chips, increased collection of partial milklike secretions containing selected bioactive components was achieved. Our work represents the successful development of a robust microphysiological system as a potential in vitro platform for mammary gland research and is promising in addressing future food and environmental crises.

## Introduction

Milk is an important source of nutrition for human infants [1–4]. The dairy farming industry plays a vital role in worldwide milk production [5,6]. Although with large-scale productivity and great commercial success, it cannot be ignored that the dairy industry has important environmental and ecological impacts such as water pollution, soil degradation, and greenhouse gas emissions [7–10]. More importantly, the global milk supply is almost entirely sourced from farm animals, which carries inherent risks of pathogen contamination, allergenicity, and antibiotic residues associated with mastitis treatment, raising safety concerns particularly for infant nutrition [11–16]. To overcome these circumstances, cellular agriculture has emerged as a promising strategy to produce animal-derived products without relying on livestock. In particular, animal-free dairy, based on cell cultivation or microbial precision fermentation, has been developed to synthesize milk components such as recombinant human lactoferrin and human milk oligosaccharides [17–19]. Despite these advances, current animal-free dairy strategies mainly focus on producing individual milk components rather than reconstructing the coordinated cellular processes underlying milk secretion [20–22]. Therefore, an in vitro platform capable of recapitulating the structural and functional features of the mammary gland is needed to generate more physiologically relevant milk bioactives’ secretions.

**Fig. 1.**
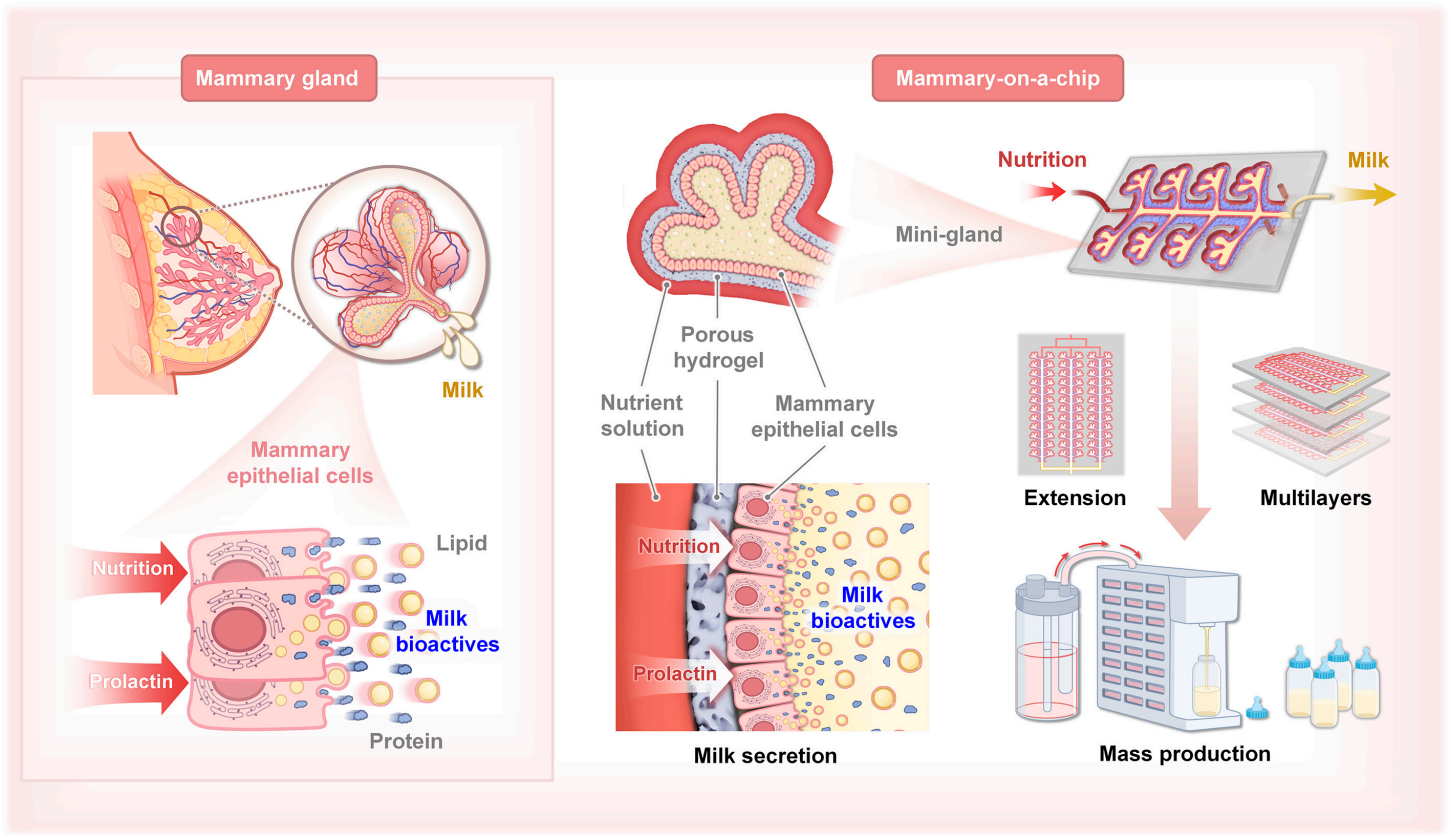
Schematic of the glandular structure of the mammary gland and the construction of the biomimetic mammary gland-on-a-chip. In the mammary gland, mammary epithelial cells (MECs) are responsible for the synthesis of milk components. The mammary-on-a-chip platform, featuring a medium channel, porous hydrogel, and specialized microchambers, recapitulate both structure and lactation. The scalable design of the chip further facilitates increased production of selected milk-associated components.

Here, inspired by the structure and function of mammary glands, we propose a mammary gland-on-a-chip system for the biomimetic production of selected human-milk-associated components, as shown in Fig. 1. Organ-on-a-chip technology has emerged as a powerful in vitro platform for recapitulating tissue microenvironments and investigating human biological functions, disease mechanisms, and drug responses [23–30]. In parallel, advances in organoid engineering, biomaterials, and microfluidic fabrication have further expanded the capability to construct physiologically relevant in vitro models [31–35]. Our system includes a vasculature-like medium channel, an intermediate porous hydrogel layer, and central alveolar-like microchambers. This configuration mimics the key physiological features of the mammary epithelium, enabling tight-junction (TJ) formation and secretion of milk bioactive components under lactation-mimicking conditions. Furthermore, primary mammary epithelial cells (MECs) were used for secretome characterization, and proteomic profiling revealed the secretion of canonical milk proteins and multiple bioactive factors overlapping with known human-milk-associated proteins. These milk components can be enriched in the central microchambers and efficiently collected [36,37]. In particular, by expanding the chambers and parallelizing the chips, scaled-up production of partial milklike secretions containing selected bioactive components could be achieved. Together, these results demonstrate that our biomimetic chip provides a controllable and scalable platform for in vitro studies of mammary gland function.

## Results

### Construction of the mammary gland-on-a-chip

**Fig. 2.**
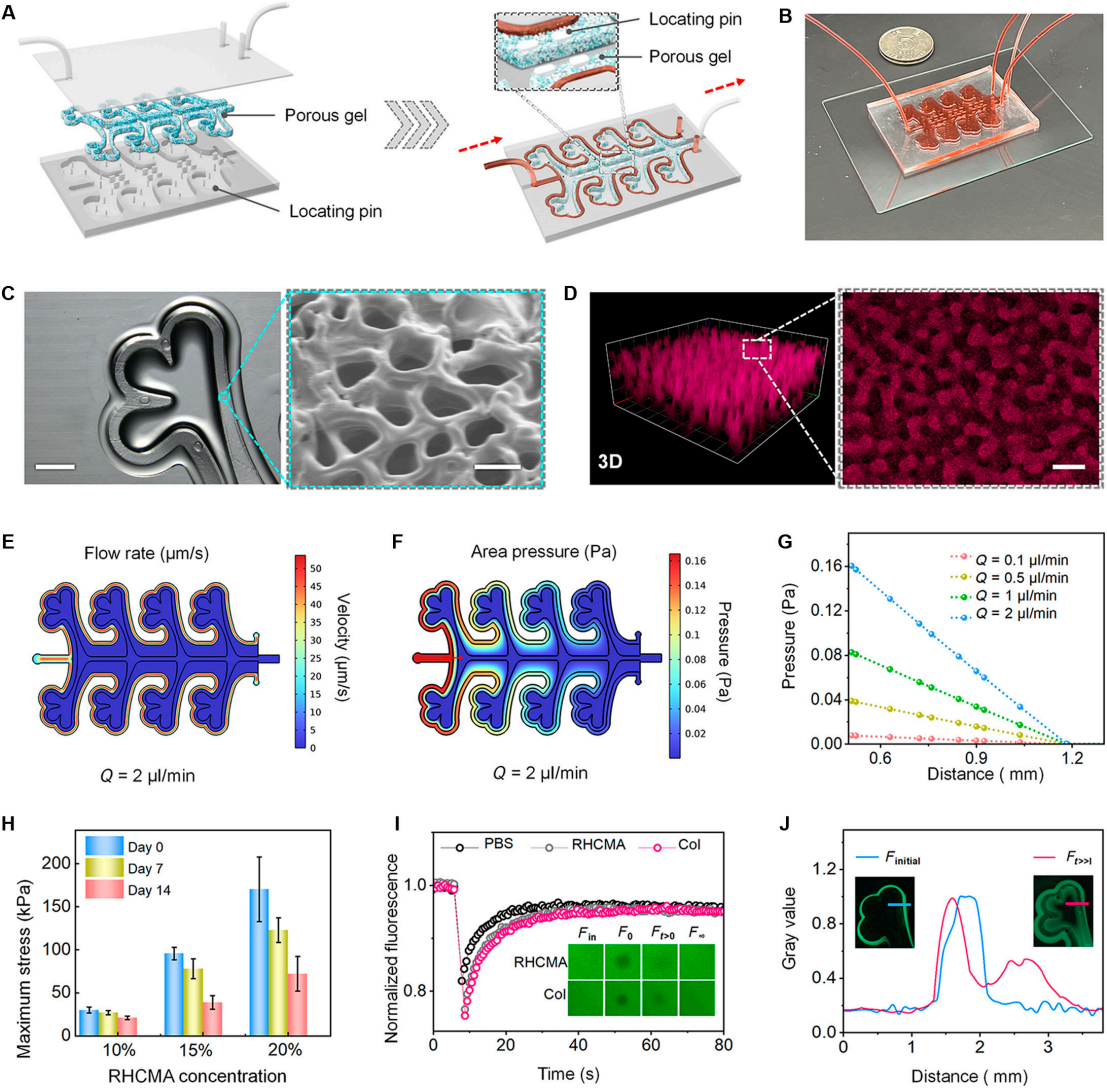
Design and characterization of the mammary gland-on-a-chip. (A) The design of the mammary gland-on-a-chip. (B) The photo of the mammary gland-on-a-chip. (C) Optical microscopic image of a basic unit of the mammary gland-on-a-chip and the scanning electron microscopy (SEM) image of the interconnected porous hydrogel. (D) The confocal laser scanning microscope image of the porous hydrogel. (E) Simulated flow velocity profile. (F) Area distribution diagram of fluid pressure. (G) Statistics of pressure distribution in the porous hydrogel. The dataset was selected from the red line segment in (F). (H) Maximum compressive stress of the hydrogels of different recombinant human collagen methacrylate (RHCMA) concentrations. (I) Normalized fluorescence recovery curve. RHCMA, RHCMA porous hydrogel; Col, RHCMA hydrogel coated with collagen. (J) Normalized fluorescence intensity profiles. The blue and pink lines in the inset images denote where the data points were taken. The scale bar is 2 mm in (C), 10 μm in the magnified image, and 20 μm in (D).

To mimic the gland structure of the mammary gland, we developed a microfluidic physiological system composed of a central channel with 8 gland microchambers arranged in parallel on both sides (Fig. 2A and B). The microchambers were designed with an alveolar-like shape and were connected to the central channel through individual ducts. The periphery of the microphysiological system was a medium channel, with a layer of interconnected porous hydrogel placed alongside. Human MECs were cultured in the microchambers. The medium channel could mimic the vasculature and allow for the efficient delivery of nutrients; the porous hydrogel could serve as a barrier separating the cells and the perfused medium, which protects cells from shear forces while allowing diffusion-based nutrient transport.

To fabricate the microfluidic chip, a polydimethylsiloxane (PDMS) mixture was utilized for replicating a customized polymethylmethacrylate template. The resulting PDMS chip was then treated with oxygen plasma and then bonded to another plasma-treated PDMS film. A mask with a predesigned pattern was employed to cure the hydrogel by ultraviolet (UV) irradiation, and then the uncured pregel solution was removed (Fig. S1). The porous hydrogel was composed of recombinant human collagen methacrylate (RHCMA), whose synthesis and characterization are shown in Fig. S2a and b. The formation of pores within the hydrogel was achieved by using polyethylene oxide (PEO) as the porogen. Briefly, a solution prepared by mixing a 20% RHCMA and a 1.6% PEO aqueous solution at a volume ratio of 9:1 was infused into the chip. To visualize the porous structure of the hydrogel, RHCMA was modified with rhodamine B, whose successful synthesis was confirmed by UV–visible spectrometry (Fig. S2c to e). We found that the pregel mixture with higher concentrations of PEO appeared black droplets with larger sizes (Fig. S3a and b). In the subsequent studies, we chose the hydrogel obtained from the abovementioned mixture at a volume ratio of 9:1, whose pore size was approximately 10 μm (Fig. 2C and D and Fig. S3c).

Numerical simulation was performed to investigate the fluid flow velocity profile in the microphysiological system (Fig. 2E and Fig. S4). The Péclet number was estimated to be 0.00065 in the microporous hydrogel. This indicated that the transport from the medium channel to the lumen area was dominated by diffusion. Furthermore, we used the fluorescence recovery after photobleaching (FRAP) assay for analyzing the distribution of fluorescein isothiocyanate (FITC)–dextran in the RHCMA porous hydrogel and that with a collagen coating. We found that the collagen coating did not significantly alter its characteristic diffusion time, as shown in Fig. 2I. To further illustrate the permeability of the porous hydrogel, a liquid containing FITC–dextran was infused through the medium channel, which could smoothly penetrate the porous hydrogel and coating layer and infiltrate into the interior of the microchambers (Fig. 2J). Moreover, the fluid pressure distribution was simulated (Fig. 2F and G and Fig. S5), and a compression test was performed, as shown in Fig. 2H and Fig. S6a and b. We found that the hydrogel can remain intact under prolonged culture conditions. Also, swelling experiments showed that the RHCMA hydrogel exhibited a less than 5% swelling ratio in phosphate-buffered saline (PBS) (Fig. S6c). These data suggested that the hydrogel can withstand dynamic deformation and maintain morphological stability during long-term culture.

### Cell–matrix interactions coordinating bilineage differentiation

**Fig. 3.**
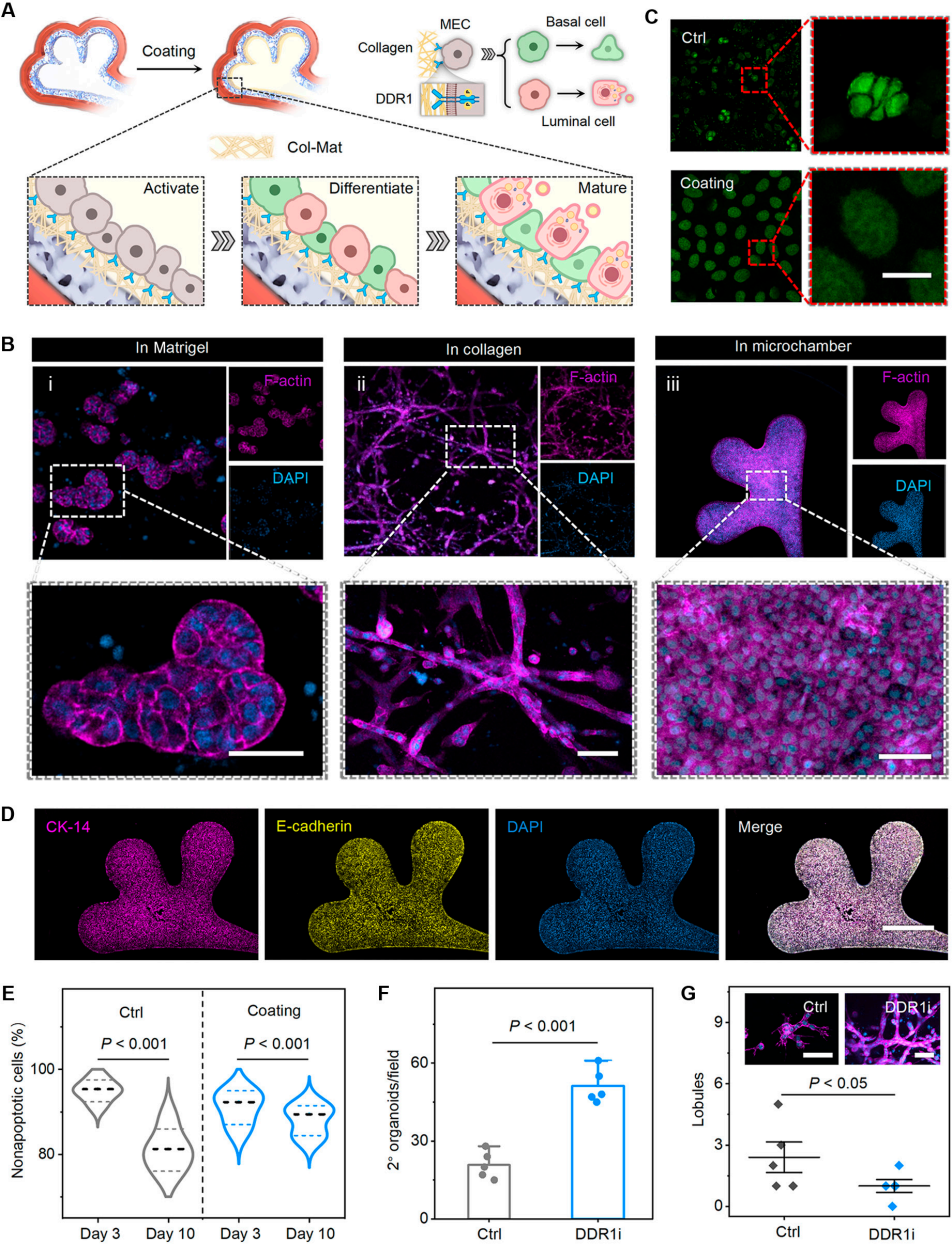
Cellular interactions and morphogenesis in the mammary gland-on-a-chip. (A) Schematic illustration of cell–matrix interactions within chambers. (B) Confocal scanning image of mammary epithelial cells (MECs) (MCF-10A) in different media, including forming acinar morphology embedded within Matrigel (i), branching morphology within type I collagen (ii), and cell sheets in mammary gland-on-a-chip (iii). (i) and (ii) are 3-dimensional (3D) layer-scanning reconstructed images. (C) Confocal scanning image showing acridine orange staining for MECs cultured on-chip with or without coating. (D) Immunofluorescence staining of luminal (E-cadherin, yellow) and basal markers (CK-14, purple) on-chip. (E) Statistical analysis of nonapoptotic MECs (*n* = 5). (F) Statistical analysis of the secondary (2°) organoid development under treatment with or without discoidin domain receptor 1 (DDR1) inhibition (*n* = 5). (G) Human breast tissue organoids’ development in hydrogels and stained by F-actin (*n* = 5). The scale bar is 20 μm in (B), 10 μm in (C), 2 mm in (D), and 50 μm in (G). Data are shown as mean ± SD. For panels (E) to (G), an unpaired 2-tailed Student *t* test was used. *n* denotes biologically independent chips or cultures, with technical image measurements averaged within each biological replicate before statistical analysis.

Mammary morphogenesis involves cell differentiation, proliferation, and self-assembly [36,37]. MECs, the primary functional cells responsible for lactation, can utilize the signal transduction components provided by the extracellular matrix to achieve self-organizing capability (Fig. 3A). For on-chip culture, the surface of the chambers was coated with Col-Mat gel (3:1), and MECs were seeded by infusion through the outlet. Similar to previous reports, we found that MECs (MCF-10A) can form different degrees of acinar-like or duct-like structures during 3-dimensional (3D) culture within different matrices (Fig. 3B (i) and (ii)). Interestingly, we found that the cells readily organized into cell sheets within the chambers (Fig. 3B (iii)). Additionally, we observed that MECs formed distinct structures when cultured “on top” of thin hydrogel layers composed of collagen–Matrigel mixtures (Col-Mat gel) of varying ratios (Fig. S7a). The corresponding Cell Counting Kit-8 cell viability assay and live staining analysis showed that the cells were able to proliferate normally over time and express high levels of F-actin (Fig. S7b to e).

The coated chambers significantly inhibited cell apoptosis for up to 10 d. In contrast, the cells showed nuclear condensation and more apoptosis on day 10 when cultured in the chambers without coating (Fig. 3C and E). Similar results were observed when the Col-Mat gel was pretreated with ammonium sulfate, which helped to remove growth factors (Fig. S8). These results indicated that the coating can extend the maintenance time of cell growth in vitro. We also found that MECs expressed stem/progenitor markers at day 1 after seeding, indicating that these epithelial cells possess differentiation potential (Fig. S9). In the mammary gland, the terminal ductal lobular units (TDLUs) consist of 2 kinds of layers: inner luminal epithelial layers and outer basal myoepithelial layers (Fig. S10). MECs seeded in the chambers for 3 d expressed both basal and luminal markers, which was similar to spheroids derived from MECs (MCF-10A) grown within 3D Col-Mat gels (Fig. 3D and Fig. S11). These results suggested that the cells grown within the microphysiological system could recapitulate selected features of their in vivo counterparts.

According to a previous study, discoidin domain receptor 1 (DDR1), responsible for collagen binding, is a regulator of breast tissue regeneration [38]. We next examined the influence of collagen on MECs’ differentiation using specific kinase inhibitors. MCF-10A cells lose their self-renewal ability during differentiation and thus cannot generate secondary tissues/organoids. Therefore, we assessed the degree of MCF-10A cell differentiation by using DDR1 inhibitors (DDR1i). MCF-10A cells were initially cultured within collagen hydrogel for 10 d to generate primary (1°) organoids that were subsequently dissociated and cultured in hydrogel to observe the formation of secondary (2°) organoids (Fig. S12a). The DDR1i treatment resulted in a significant increase in 2° organoid formation, suggesting a reduced degree of differentiation (Fig. 3F). Furthermore, DDR1i treatment had no significant effect on detectable apoptotic changes (Fig. S12b). These findings indicated the importance of collagen in mammary morphogenesis.

To explore DDR1’s involvement in differentiation, primary cell clusters (organoids) isolated from the tissue of reduction mammoplasty patients were seeded within the collagen hydrogel. After treatment with DDR1i, ducts formed, but no budding or lobule development occurred, suggesting that DDR1 is important for TDLU formation (Fig. 3G). Additionally, a mammosphere assay was conducted to examine the stem/progenitor activity by culturing primary organoids for 14 d with or without DDR1i and then examining the colony formation in suspension (Fig. S13a). We found that with DDR1i treatment, cells extracted from hydrogel culture produced a larger number of mammospheres compared to the untreated group (Fig. S13b), indicating that DDR1i affects the function of stem/progenitor cells rather than their abundance. Additionally, DDR1i treatment had no significant impact on the luminal/basal cells’ ratio (Fig. S13c and d). These results validated the role of cell–matrix interaction in coordinating the bilineage differentiation and provided support for the use of the Col-Mat gel in the construction of the microphysiological system.

### Milk bioactive ingredient secretion

**Fig. 4.**
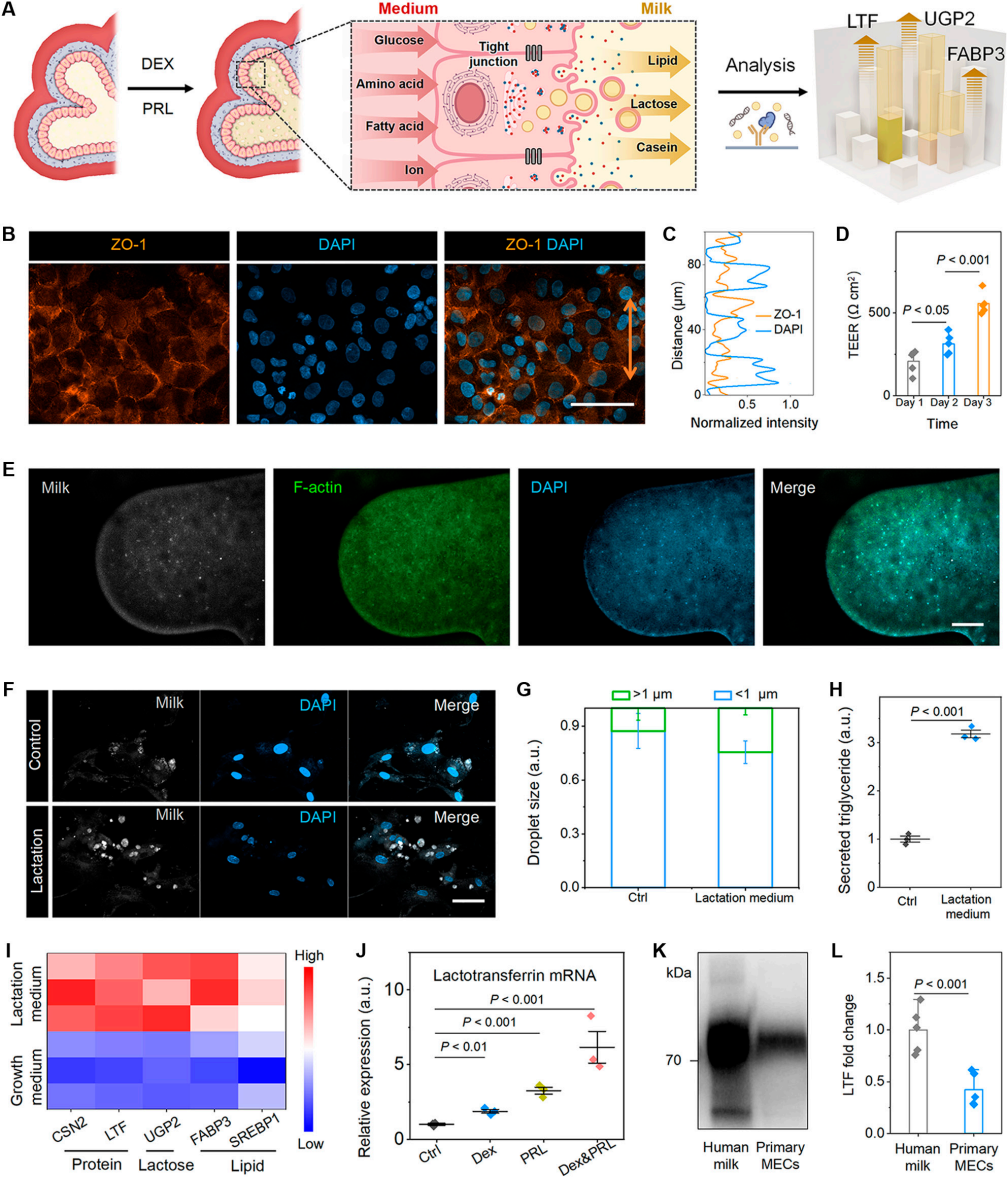
Lactation and tight-junction (TJ) formation in the mammary gland-on-a-chip microphysiological system. (A) Schematic diagram of lactation and TJ formation in the microphysiological system. (B) TJs in primary mammary epithelial cells’ (MECs’) growth on-chip with zonula occludens-1 (ZO-1) labeled in orange and 4′,6-diamidino-2-phenylindole (DAPI) in blue. (C) Statistical distribution of normalized fluorescence intensity. The orange line represents where the data were taken. (D) Transepithelial electrical resistance (TEER) between the medium channel and the chamber in 3 d (lactation culture period) (*n* = 5). (E) Nile Red staining (lipid droplets, white) and F-actin staining (cytoskeleton, green) of primary MECs on-chip after lactation medium stimulation. (F) Nile Red staining images of primary MECs on-chip before and after lactation medium stimulation. (G) Statistics of size distribution of the lipid droplets (*n* = 3). (H) Relative amount of triglyceride secretion (*n* = 3). (I) Heatmap of messenger RNA (mRNA) expression related to lactation in MECs, including CSN2, LTF, UGP2, FABP3, and SREBP1. (J) Relative expression levels of LTF mRNA in primary MECs cultured on-chip under different treatments (*n* = 3). (K) Western blot (WB) analysis of LTF. (L) Relative WB band intensity (*n* = 5). The scale bar is 50 μm in (B) and (F) and 300 μm in (E). Data are shown as mean ± SD. One-way analysis of variance (ANOVA) with Tukey’s post hoc test was used for panels (D) and (J); an unpaired 2-tailed Student *t* test was used for panels (H) and (L). *n* represents biological replicates.

We used primary MECs isolated from human tissues for investigating milk bioactive ingredients’ secretion (Fig. S14). In the lactating mammary gland, luminal MECs serve as secretory cells for lactation. To produce major milk bioactive ingredients on-chip, the chambers were supplied with growth medium for the first 3 d, followed by lactation medium containing dexamethasone (Dex) and prolactin (PRL) to stimulate milk production (Fig. 4A). We found that primary MECs cultured on-chip expressed representative TJ proteins, similar to that in breast tissue (Fig. 4B and C and Fig. S15). Additionally, we measured the transepithelial electrical resistance (TEER) between the medium channel and the chamber under different medium conditions. A significant increase in TEER was observed in the lactation medium group on day 2, suggesting improved TJ integrity (Fig. S16). Moreover, under the lactation medium treatments, the TEER increased with time (Fig. 4D).

Next, we investigated the function of milk secretion of the microphysiological system. We first tested the efficiency of the lactation medium in primary mammary organoids. Nile Red staining results revealed that the lactation medium induced the synthesis of abundant lipid droplets within the organoids, indicating that the lactation medium can induce and promote lactation in mammary organoids (Fig. S17). Then, the synthesis of lipid droplets was also detected in the mammary microphysiological system (Fig. 4E). We found that the stimulation of the lactation medium increased the quantity of lipid droplets (Fig. 4F to H). We further measured the expression of milk-related messenger RNAs (mRNAs) in primary MECs cultured on-chip and found that the mRNAs involved in protein (β-casein [CSN2]), lactotransferrin (LTF), lactose (UGP2), and triglyceride syntheses (FABP3 and SREBP1) were upregulated to varying degrees (Fig. 4I). We next investigated the roles of Dex and PRL by utilizing different formulated culture media. A single treatment with Dex or PRL (in a medium containing only Dex or only PRL, respectively) increased the expression of CSN2 and LTF. The combined treatment (Dex and PRL) showed the highest expression level in CSN2 and LTF (Fig. 4J and Fig. S18a). In addition, all treatment groups promoted secreted triglyceride level (Fig. S18b). Moreover, we found that organoids cultured in Matrigel led to secreted proteins accumulating intracellularly or being trapped within acinar-like structures. In contrast, there was minimal accumulation of secreted proteins in primary MECs cultured on-chip (Fig. S19a). Quantitative analysis tests also confirmed this phenomenon (Fig. S19b and c), indicating that the mammary gland-on-a-chip enables efficient collection of secreted milk components.

We next collected the secreted medium from the outlet of the central perfusion channel after 5 d of lactation medium stimulation and compared it with human milk by proteomics. In total, 256 proteins were identified in human milk and 360 in the primary MECs’ secretome, with 80 shared between the 2 (Fig. S20a). Within the secreted subproteome, 141 and 166 proteins were detected in human milk and the MEC secretome, respectively (Fig. S20b). Gene Ontology analysis for total proteins revealed strong concordance between groups, with top terms mainly related to extracellular and vesicle-associated processes (Fig. S20c and d). Western blot further confirmed LTF expression in the secreted medium, although at lower levels than those in human milk (Fig. 4K and L). Moreover, both primary MECs and epithelial cell lines (MCF-10A and MAC-T) produced milk components and lipid droplets on-chip, confirming the lactation functionality of the system (Fig. S21). These results demonstrate that the microphysiological system effectively supports milk production.

### Scaled-up production of selected milk bioactive components

**Fig. 5.**
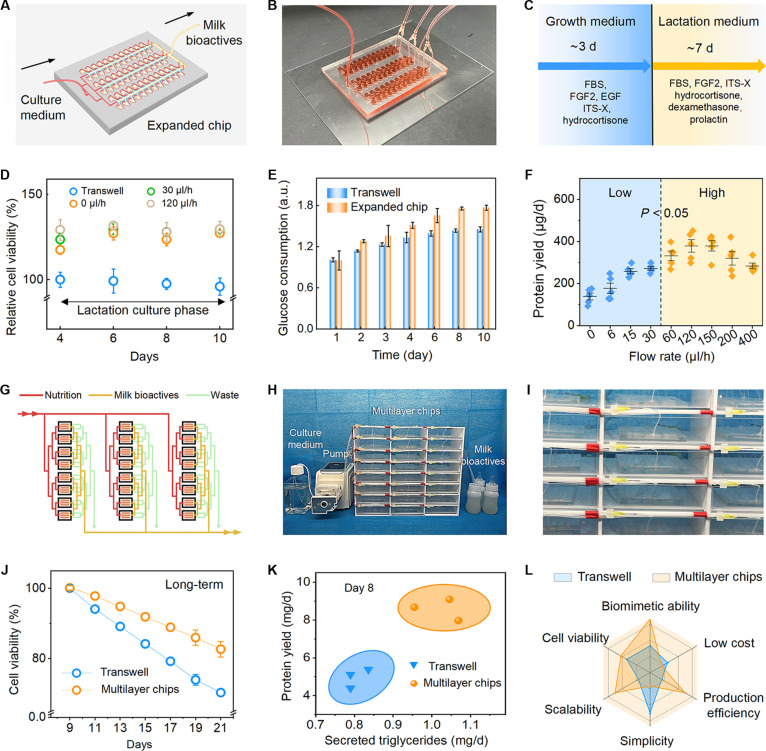
Comprehensive evaluation of the expanded microfluidic chip and the integrated mammary microphysiological system. (A) Schematic illustration of the expanded chip. (B) Photograph of the expanded chip. (C) Illustration of the growth culture phase and secretion culture phase and their corresponding medium components. (D) Effect of flow rate on primary mammary epithelial cells’ (MECs’) viability during the lactation phase on the expanded chip (*n* = 5). For the Transwell group, primary MECs were seeded into the upper chamber, whose surface was coated with the same Col-Mat hydrogel as that in the chip; the medium in the lower chamber was identical to that used in the chip group. (E) Glucose consumption of primary MECs on the expanded chip and in Transwell (*n* = 5). (F) Effect of flow rate in the medium channel on total protein yield on day 10 in the expanded chip (*n* = 5). (G) Schematic diagram of the scaling-up of the integrated mammary microphysiological system. (H) Photograph of the integrated mammary microphysiological system. (I) Magnified image of multilayer chips. (J) Statistical analysis of cell viability within the chip during long-term culture. (K) Comparison of the protein yield and triglyceride yield of different groups. (L) Comparison of the merits between Transwell system and the multilayer chips. Data are shown as mean ± SD. One-way analysis of variance (ANOVA) with Tukey’s post hoc test was used for panel (F); *n* represents biological replicates.

Building on the design of the 8-chamber chip described above, we developed an expanded chip containing 72 chambers. As a control, a conventional Transwell culture system was used to compare cell viability and secretory performance. The first 3 d were the growth culture phase, and the last 10 d were the secretion culture phase, as illustrated in Fig. 5A to C. We further optimized the effect of flow rate in the medium channel on milk bioactive production. The flow rate during the growth culture phase showed a positive correlation with cell viability and viable cell count (Fig. S22). The primary MECs’ viability on the expanded chip at different flow rates during the lactation culture phase was consistently higher than that of the Transwell system (Fig. 5D). Glucose consumption during both culture phases was higher than that observed in the Transwell group (Fig. 5E). Additionally, we found that the flow rate also affected the total protein yield, and we set 60 μl/h as the optimized condition (Fig. 5F). Compared to the Transwell model, the expanded chip achieved significantly higher protein and triglyceride levels during 12 d of culture (Fig. S23). Notably, prolonged cell culture led to a decline in total protein and triglyceride yields, probably due to the onset of partial apoptosis of the cells.

To increase production at a laboratory-chip scale, the expanded chip was scaled up to a multilayer configuration and an integrated system was set up. Specifically, 24 expanded chips were connected in series. Each chip’s inlet was supplied with the culture medium through a peristaltic pump, while another peristaltic pump at the outlet collected the secreted medium into a designated collection device. This ensures continuous and efficient operation for enhanced scalability (Fig. 5G to I and Fig. S24). The cell viability on the multilayer chip system and the secretion ability of cells in the lactation culture phase were found to be maintained for an extended period (Fig. 5J and Fig. S25). The total protein and triglyceride production reached approximately 8.57 and 1.02 mg per integrated multichip system per day, respectively, in the integrated chip system. These values were significantly higher than those obtained from the Transwell culture system with the same culture area (Fig. 5K). The multilayer chips have several significant advantages, including biomimetic ability, low cost, high cell viability, high production efficiency, scalability, and simplicity (Fig. 5L). These findings suggested that the present mammary microphysiological system achieves scale-up production of milk bioactive substances in vitro by simulating real mammary physiological conditions.

## Discussion

In summary, we have developed a biomimetic mammary gland-on-a-chip platform for modeling selected structural and functional features of the human mammary gland and collecting secreted milk bioactive components in vitro. Our mammary gland-on-a-chip platform integrates a medium channel, interconnected porous hydrogels, and central gland microchambers to re-establish key physiological processes, including bilineage differentiation of MECs driven by collagen-activated DDR1 signaling, the formation of epithelial networks and TJs, and secretion of milk bioactives like lactotransferrin, β-casein, and triglycerides. The specialized design of the chip also enables the enrichment and collection of milk components from within the central microchambers. Furthermore, by parallelizing multiple mammary gland-on-a-chip units, the system allows for scalable production of milk ingredients with complicated higher-order structures, providing a potential platform for the scalable in vitro production of selected milk bioactive components.

The integration of the mammary gland biology and advanced organ-on-a-chip technology establishes a system with distinctive advantages. Unlike other traditional culture systems, the mammary gland-on-a-chip can recapitulate selected aspects of tissue organization and lactation-related secretion under controllable perfusion conditions, which may also have been overlooked in other types of lab-grown milk production techniques. Future efforts may focus on integrating optimized sensors on-chip to detect even subtle biological signals, thus translating cellular behavior, such as metabolite alterations, into measurable outputs and providing feedback to the regulation of the operating parameters. In addition, with further refinement of the mammary gland-on-a-chip and possible employment of multiorgan-on-a-chip, we expect to produce milk ingredients with complicated, higher-order structures in a biomimetic manner. Equally important, future studies should incorporate biosafety validation and purification processes to ensure that the secreted biofluids are free of residual materials (e.g., matrix components) and meet the safety standards required for downstream applications. Moreover, the system’s scalability may be further improved to meet commercial needs, and a critical step toward this goal is to ensure batch consistency and long-term milk production. With that, we believe that the mammary gland-on-a-chip holds potential for advancing human milk research, supporting cell-based production of selected milk-associated components, and addressing global challenges in biotechnology and food production.

## Methods

### Characterization of hydrogel swelling and mechanical properties

The compression test of RHCMA hydrogels were conducted under a 10 mm/min loading rate. Cylindrical hydrogel samples were immersed in PBS for 60 min (day 0), 7 d, and 14 d before testing. Stress–strain results were generated, and the maximum compressive stress was recorded. For swelling ratio analysis, cylindrical hydrogel samples (2 mm in height) were prepared and equilibrated in PBS at 37 °C for 24 h. After measuring their wet weight, the samples were frozen at −80 °C for 2 h and lyophilized overnight, and the swelling ratio was determined by comparing wet and dry weights.

### Construction of the mammary gland-on-a-chip

Briefly, a PDMS (Corning) mixture (10:1 w/w ratio of base to hardener) was utilized for replicating a customized polymethylmethacrylate template with defined microchannel patterns. The medium perfusion channels were 300 μm in width and 1 mm in height. The resulting PDMS chip was exposed to oxygen plasma (100 W, 1 min, under 0.6 mbar) and then irreversibly bonded to another plasma-treated PDMS film. To load and cure the hydrogel, a 1.6% PEO solution (prepared in PBS) and a 20% RHCMA solution (in deionized water) were mixed at a 1:9 volume ratio under gentle stirring for 5 min. The pregel solution was gently infused into the microchamber through the inlet and allowed to sit at 37 °C for 5 min. The hydrogel layer thickness was approximately 500 μm, with minor variation depending on the projection and fabrication process. Subsequently, after aligning the mask under a stereomicroscope, the RHCMA portion was exposed to UV radiation (365 nm, 10 mW/cm^2^) to cure it. PBS solution (10 ml) was infused through the loading ports and flushed repeatedly to remove uncured pregel solution and residual PEO until the eluent became clear.

### Characterization of diffusion

FITC–dextran (1 mg/ml, Sigma-Aldrich) [39] in PBS was introduced into the microfluidic chip at 60 μl/h, and fluorescence distribution images were captured after 10 min. FRAP experiments were performed using a confocal microscope (980 Zeiss) with prebleach scans at full intensity, followed by monitoring recovery with low-intensity scans. Fluorescence intensities were normalized to prebleach levels.

### Simulation on the flow rate and pressure

The flow rate and pressure on-chip were simulated using COMSOL Multiphysics (version 6.1), with hydrogel regions modeled as porous media [40,41]. The simulations were based on the Navier–Stokes equations, assuming that the flow was homogeneous. A dynamic viscosity of 0.692 mPa·s and a density of 1 g/ml were set to simulate the culture medium. The outlet pressure was 0 Pa, and steady-state, fully developed flow was assumed with no-slip boundary conditions. The Péclet number ($P_{e}$) was calculated using the formula

$$P_{e}=\frac{U\cdot L}{D}$$ (1)

where *U* represents the average velocity, *L* is the characteristic length, and *D* denotes the diffusion coefficient.

### Cell culture

MCF-10A cells were purchased from Wuhan Sunncell Biotechnology Co., Ltd. Primary human MECs were isolated from reduction-mammoplasty-derived mammary tissues obtained from nonlactating donors. Briefly, fresh tissue specimens were washed with antibiotic-containing PBS buffer, and visible fat and connective tissue were carefully removed. The remaining mammary tissue was mechanically minced into small fragments and gently digested with agitation for 3 h at 37 °C in Dulbecco’s modified Eagle medium (DMEM)/F12 medium containing collagenase (NeoFroxx). After digestion, the supernatant was removed by centrifugation, and the precipitate was resuspended in DMEM/F12 medium containing 0.5% trypsin (Thermo Fisher) and incubated for 3 min to further dissociate tissue fragments. Fetal bovine serum (Gibco) was added to terminate digestion, followed by centrifugation at 700 g for 3 min. The precipitate was resuspended in 50% fetal bovine serum and centrifuged at 1,000 g for 3 min. This differential centrifugation step was repeated to enrich the mammary epithelial fragments and reduce contamination by stromal and fatty components. The isolated epithelial fragments were then resuspended in breast epithelial cell culture medium and seeded for amplification in preparation for subsequent experiments. Epithelial characteristics were confirmed by the cobblestone-like morphology of the isolated cells and the expression of breast epithelial markers.

For 3D cultures, the cell suspension was mixed with Matrigel (Corning), type I collagen (Corning), or Col-Mat hydrogel at a 1:1 volume ratio, followed by gentle mixing at 4 °C to ensure homogeneity. The hydrogel–cell mixture was then injected into the well plate and incubated for about 1 h to gelation in a CO_2_ incubator.

For matrix–surface cultures, Matrigel and type I collagen were mixed at defined volume ratios (1:3, 1:1, and 3:1), followed by seeding of equal volumes of cells at 2 × 10^4^ cells per well.

For cell perfusion within the chip, a cell suspension at 5 × 10^5^ cells/ml was introduced by gravity-driven flow [42] to fill the entire mammary microchamber.

### Apoptosis detection

For apoptosis detection, the chip was cut around the hydrogel chamber, and the chamber was rinsed with PBS for about 10 s, after which the PBS was removed. Acridine orange staining solution (Beyotime Biotechnology) was added. Green fluorescence was observed under a confocal microscope (Zeiss). For flow cytometry analysis, cells were digested from the chip at various time points.

## Ethical Approval

The use of human mammary tissues was approved by the IEC for Clinical Research of Zhongda Hospital, Affiliated to Southeast University (Approval No. 2025ZDSYLL044-P01).

## Data Availability

The data supporting the findings of this study are available in the article and its supplementary materials.
