# Supplementary material for: Biomimetic Mammary Gland Organoid-on-a-Chip for Producing Selected Human Milk Components
Source: Research (Wash D C). 2026 Jul 17;9:1356. doi: 10.34133/research.1356 (PMC13376379; doi:10.34133/research.1356)
Supplement: Supplementary 1 — Supplementary Methods Figs. S1 to S25 Table S1 [file research.1356.f1.docx]

**Supplementary Methods**

***Synthesis of RHCMA:*** RHCMA (Shanxi Jinbo Bio-Pharmaceutical, China) was synthesized following an established protocol^1^. In brief, lyophilized RHC was dissolved in PBS, and methacrylic anhydride (MA, Sigma-Aldrich) was gradually introduced in excess. Upon completion, the reaction was halted, and the product underwent dialysis in deionized water for five days, followed by lyophilization for preservation. The structural characteristics and MA modification of RHCMA were examined using ¹H NMR spectroscopy on a spectrometer (Bruker 600 MHz, Germany).

***Preparation of RB-RHCMA:*** To prepare the RB-RHCMA conjugate, RHCMA was dissolved in deionized water at an elevated temperature, followed by the gradual addition of an activation solution containing Rhodamine B, NHS (N-Hydroxysuccinimide), and EDC (1-Ethyl-3-(3-dimethylaminopropyl) carbodiimide) in DMF (Dimethylformamide). The mixture was stirred under controlled conditions to facilitate the reaction. The resulting solution was then divided into dialysis bags (14.5 kDa). Dialysis was carried out at 37°C for three days, with the external solution replaced four times daily. After dialysis, the contents were frozen and lyophilized for three days to obtain RB-RHCMA, which was then stored under the same low-temperature conditions. To study phase separation between PEO (Polyethylene oxide, Sigma-Aldrich) and RHCMA, a 1.6% PEO solution in PBS was prepared and mixed with a 20% RB-RHCMA solution at varying ratios.

***Effect of DDR1i:*** MECs were embedded in collagen and cultured in well plates with or without DDR1i for 3 days. Apoptosis was assessed using the Annexin V FITC apoptosis kit (Biyuntian Biotechnology).

***Colony and mammosphere formation assays:*** Primary mammary organoids were cultured for 14 days with or without DDR1 inhibitor (2 μM, Tocris Bioscience). Afterward, the tissue was dissociated using 100 μg/mL collagenase in PBS. The MECs suspension was then centrifuged (300×g for 3 minutes), and the cells were resuspended. For colony formation, cells were plated and cultured for 7 days. For mammosphere formation, cells were cultured for 14 days.

***3D mammary organoid culture and staining:***Primary organoids were combined with Col-Mat gel and plated into 24-well plates^2-4^. After solidification, culture medium was added. The growth medium consisted of DMEM/F12 (Gibco), supplemented with bovine serum albumin (BSA, Sigma-Aldrich), ITS-X (Thermo Fisher), epidermal growth factor (EGF, Sinobiological), and phosphatidylethanolamine (Sigma-Aldrich). For secretion medium, the medium included oleic acid (200 µM, Sigma-Aldrich), prolactin (1 µg/mL, STEMCELL Technologies), and DEX (100 nM, Sigma-Aldrich). Organoids were fixed and stained with Nile Red (Biyuntian Biotechnology). Afterward, they were incubated with DAPI and Phalloidin-AlexaFluor 488 for 1 hour.

***Mammary microphysiological system culture and secretion detection:***Primary MEC cell sheets were digested and seeded into the microchambers of the microfluidic chip as previously described. Growth medium was perfused through the chip for the first three days, after which it was replaced with the lactation-inducing medium. Lactoferrin content was detected using a lactoferrin ELISA kit (Jiangsu Meimian Co., Nanjing, China). To perform Oil Red O staining (Biyuntian Biotechnology) on the cells within the chip, the excised microchambers were stained following the same steps used for Nile Red staining described earlier. The milk fat (Triglyceride) amount was measured using the Triglyceride Assay Kit (Biovision).

***Total Protein extraction and quantification:***Human milk was obtained from healthy lactating donors at Zhongda Hospital, Southeast University, under institutional ethical approval. After natural thawing at 25 °C, milk samples were centrifuged (10,000 × g, 30 min) to remove the lipid layer, and the aqueous fraction was used for protein analyses.

Secreted medium from chip cultures was cleared by low-speed centrifugation (1,000 × g, 10 min) to eliminate debris and directly subjected to protein quantification using a BCA kit (Solarbio). Intracellular proteins were extracted from cultured cells with TRIzol reagent (Invitrogen) following complete lysis, and clarified lysates were collected for analysis. Total protein values were corrected using cell-free lactation medium and hydrogel-conditioned medium processed under the same conditions. Collected samples were centrifuged at 1,000 × g for 10 min before analysis to minimize contributions from cell debris.

***Sample processing for proteomics and Western blotting:*** For proteomic assays, milk protein fractions were adjusted to pH ≈ 4.7 with HCl, incubated at 40 °C to precipitate casein, and centrifuged; the supernatant (whey fraction) was processed for further analysis. Secreted proteins were enriched by TCA precipitation (10%, −20 °C, 2 h), washed with cold acetone, and solubilized in urea buffer prior to tryptic digestion. For Western blotting, 20 µg proteins were separated by SDS–PAGE and probed with antibodies against lactoferrin (LTF, 1:1000) followed by HRP-linked secondary antibody. To avoid overloading due to high protein content, milk samples were diluted ten-fold before electrophoresis.

***qPCR analysis:*** Total RNA from cell layers was isolated with TRIzol reagent and reverse-transcribed using the PrimeScript RT kit (Takara). qPCR was performed with SYBR Green reagents on a StepOnePlus system (Applied Biosystems), and relative expression was calculated by the 2^−ΔΔCt^ method using GAPDH as reference. Primer sequences are provided in **Table S1**.

***Proteomic analysis:*** Samples were lysed in a buffer containing 4% SDS, 0.1 M Tris-HCl (pH 8.0), and 0.1 M DTT, heated for 7 min, and centrifuged to remove insoluble debris. The protein-containing supernatants were collected and subjected to sequential enzymatic digestion with Lys-C at an enzyme-to-protein ratio of 1:20 (w/w), followed by trypsin at an enzyme-to-protein ratio of 1:50 (w/w). The resulting peptides were desalted using C18 solid-phase extraction columns and reconstituted in 2% acetonitrile and 0.1% formic acid before LC-MS/MS analysis. LC-MS/MS analysis was performed at the Analytical Testing Center of Southeast University using an EASY-nLC 1200 nanoLC system coupled to an Orbitrap Eclipse Tribrid mass spectrometer ( Thermo Fisher Scientific). Peptides were separated on a reversed-phase C18 analytical column using mobile phase A, 0.1% formic acid in water, and mobile phase B, 0.1% formic acid in acetonitrile. Peptide separation was performed using a 60-min gradient from 5% to 35% mobile phase B. The mass spectrometer was operated in positive electrospray ionization mode. Data were acquired in data-dependent acquisition mode. Full MS scans were acquired in the Orbitrap analyzer, and the most abundant precursor ions were selected for tandem MS analysis. MS/MS spectra were generated by higher-energy collisional dissociation fragmentation and acquired for peptide sequence identification.

Raw LC-MS/MS data were processed using MaxQuant software and searched against the UniProt Homo sapiens protein database. Trypsin/P was specified as the digestion enzyme, with up to two missed cleavages allowed. Carbamidomethylation of cysteine was set as a fixed modification, whereas methionine oxidation and protein N-terminal acetylation were set as variable modifications. The precursor ion mass tolerance and fragment ion mass tolerance were set according to the default parameters for Orbitrap high-resolution MS/MS data in MaxQuant. The false discovery rate (FDR) was controlled at 1% at the peptide-spectrum match, peptide, and protein levels using a target-decoy strategy. Only proteins identified with at least one unique peptide were retained for downstream analysis. Protein quantification was performed using the label-free quantification (LFQ) algorithm in MaxQuant. Functional enrichment analysis of differentially expressed proteins was conducted using DAVID Bioinformatics Resources.

***Immunofluorescence staining:***For staining of mammary organoids or MECs cell sheet, the samples were chemically fixed in a mixture of 4.0% paraformaldehyde (PFA, Servicebio) for 15 minutes. After two washes with PBS, the organoids were stained with 5 µg/mL FITC-phalloidin (Biyuntian Biotechnology) for 60 minutes, and washed twice again with PBS. Nuclear staining was performed with DAPI for 10 minutes, followed by two washes with PBS. Excess moisture was removed, and the samples were observed under a confocal microscope. For on-chip samples, the microfluidic chip was cut around the hydrogel chamber. The antibodies used were: EpCAM (1:100, 84073-6-RR, Proteintech), CD44 (1:100, CL594-15675, Proteintech), CK6 (1:100, 10590-1-AP, Proteintech), Muc1 (1:100, 83311-4-RR, Proteintech), CK8 (1:100, GB15231-100, Servicebio), Vimentin (1:100, MA5-11883, Thermo Fisher ), LTF (1:50, PA1-29287, Thermo Fisher) and CK14 (1:100, MA5-11599, Thermo Fisher). After incubation, the samples were conjugated with corresponding secondary antibodies (Thermo Fisher) and imaged.

***Statistics:*** All quantitative data are presented as mean ± standard deviation (SD). Quantitative analyses were performed using at least three biological replicates per group. For comparisons between two independent groups, an unpaired two-tailed Student’s t-test was used. For comparisons involving multiple groups, different time points, or varying flow rates, one-way or two-way analysis of variance (ANOVA) was performed. A value of p < 0.05 was considered statistically significant.

**References:**

1. Lin, X., Filppula, A.M., Zhao, Y., Shang, L. & Zhang, H. Mechanically regulated microcarriers with stem cell loading for skin photoaging therapy. *Bioactive Materials* **46**, 448-456 (2025).

2. Charifou, E., Sumbal, J., Koledova, Z., Li, H. & Chiche, A. A Robust Mammary Organoid System to Model Lactation and Involution-like Processes. *Bio-protocol* **11**, e3996-e3996 (2021).

3. Sumbal, J., Chiche, A., Charifou, E., Koledova, Z. & Li, H. Primary Mammary Organoid Model of Lactation and Involution. *Frontiers in Cell and Developmental Biology* **8** (2020).

4. Yuan, L. et al. Reconstruction of dynamic mammary mini gland in vitro for normal physiology and oncogenesis. *Nature Methods* **20** (2023).

**Supporting Figures:**


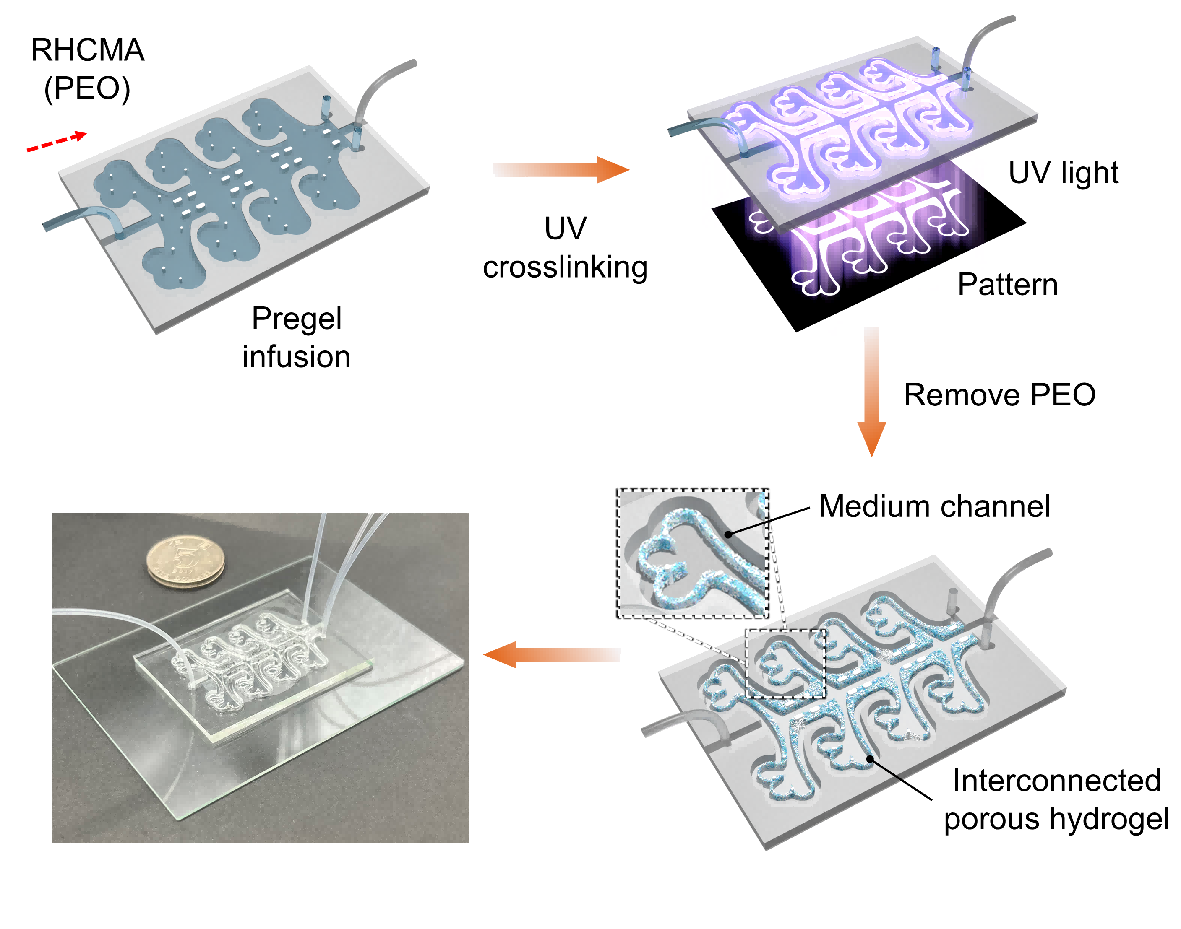


**Figure S1.** **The preparation process of the porous hydrogel and the photograph of the resultant chip.**


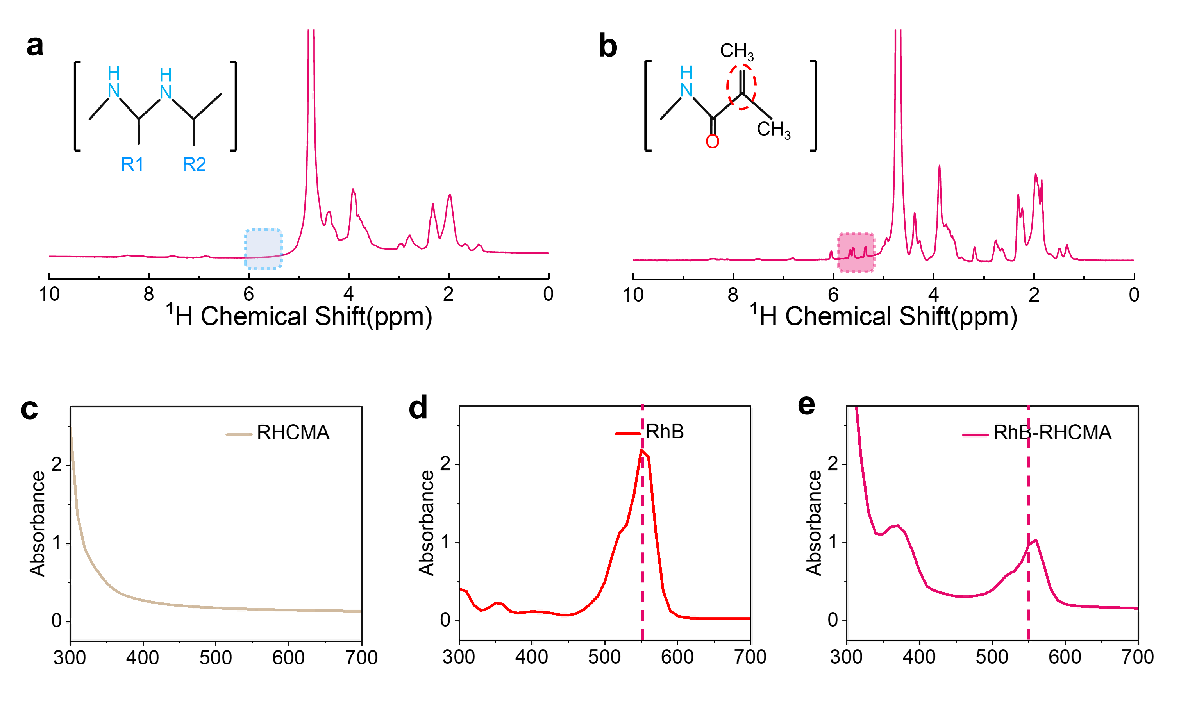


**Figure S2.** **Characterization of recombinant human collagen.**^1^H NMR spectra of a) recombinant human collagen and b) photo-crosslinkable methacrylated recombinant human collagen. c-e) UV-Vis absorption spectra recorded at 300-750 nm for RHCMA, Rhodamine B (Rh B), and RhB-RHCMA.


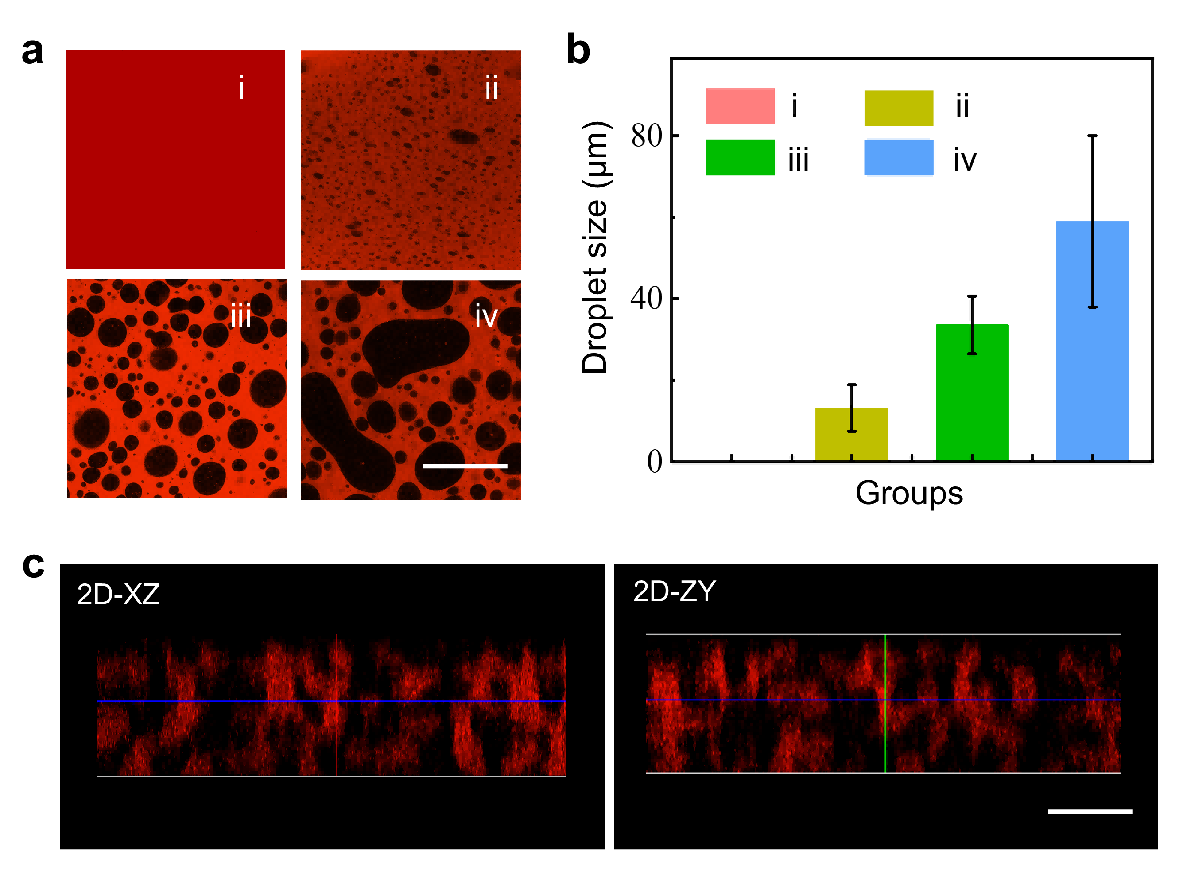


**Figure S3.** **Analysis of the morphological properties of the porous hydrogel prepared under various conditions.** a) Fluorescence images of the pregel solution of 20% RHCMA and 1.6% PEO (containing 0.5% LAP) mixed at different volume were tested with ratios of 10:0 (i), 9:1 (ii), 7:3 (iii), and 1:1 (iv). b) Statistical analysis of PEO droplet sizes in different groups (n=5). c) Confocal 3D reconstruction images of the porous hydrogel derived from 20% RHCMA and 1.6% PEO (containing 0.5% LAP) mixed at volume ratio of 9:1. The scale bar is 100 μm in a) and 20 μm in c).


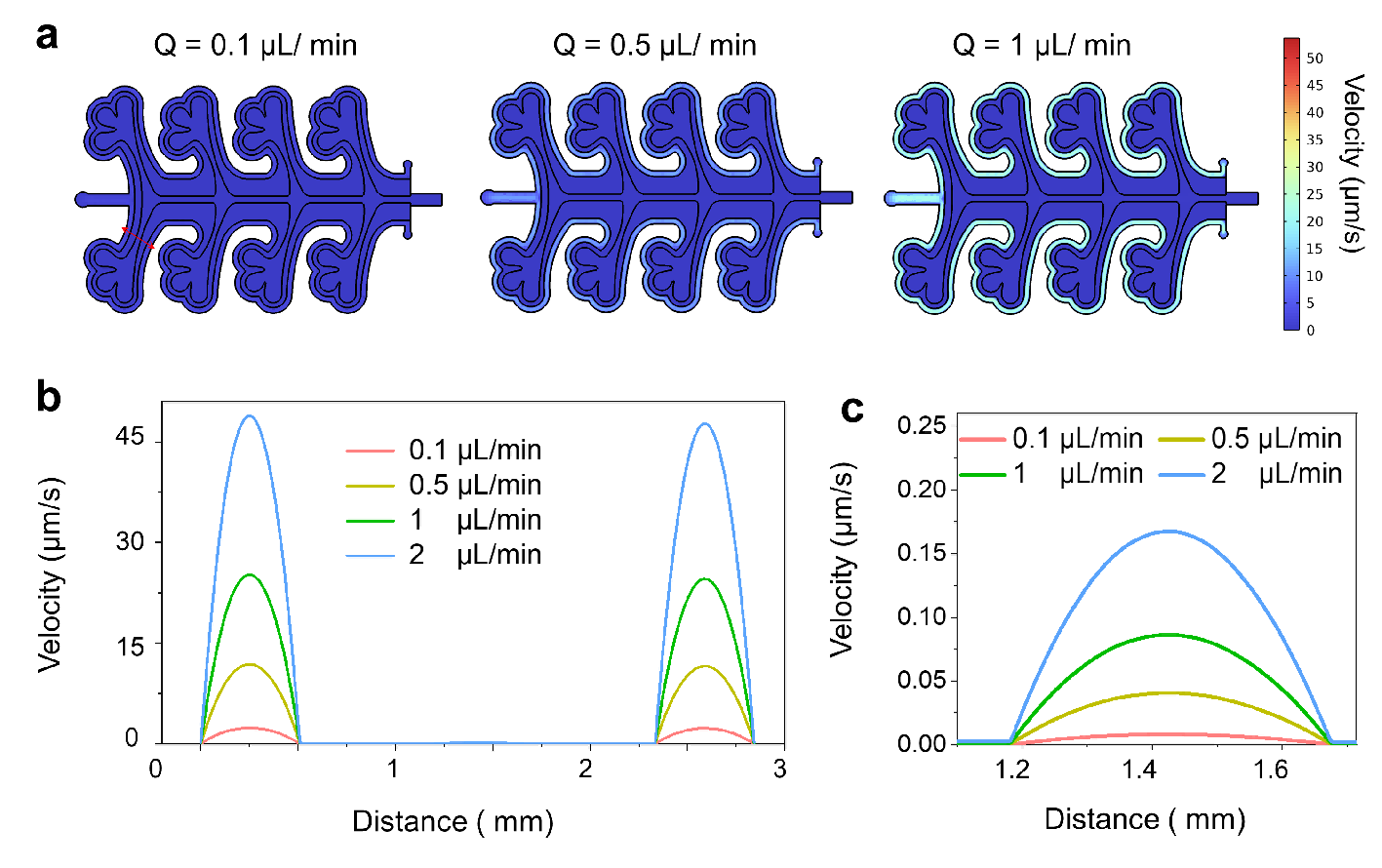


**Figure S4.** **Simulation of fluid velocity distribution.** a) Simulation of fluid velocity distribution at different inlet flow rates. b-c) Corresponding velocity distribution. The dataset was selected from the red line segment in a).


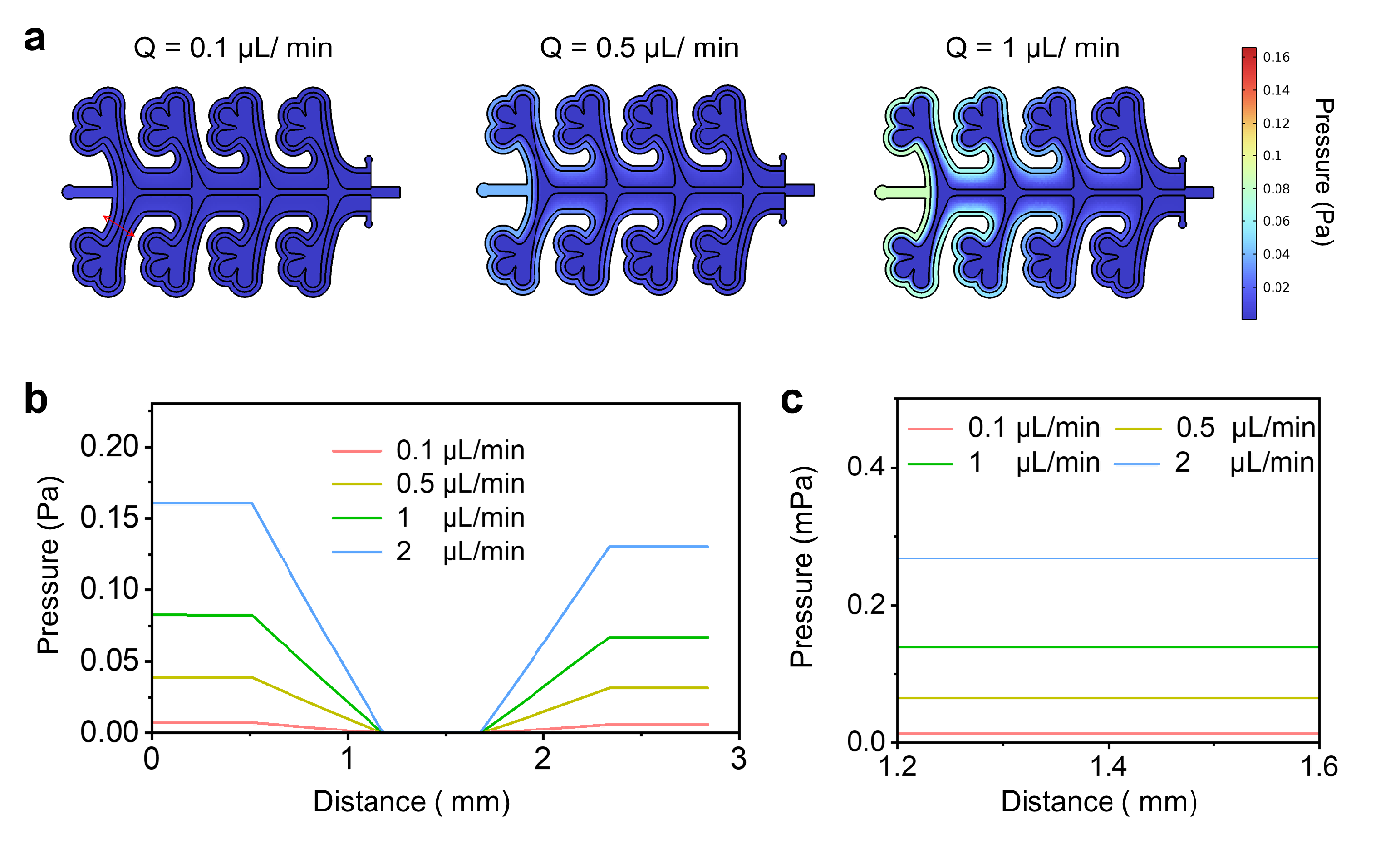


**Figure S5.** **Assessment of fluid pressure distribution.** a) Fluid pressure distribution in porous hydrogels at different inlet flow rates. b-c) Corresponding pressure distribution. The dataset was selected from the red line in a).


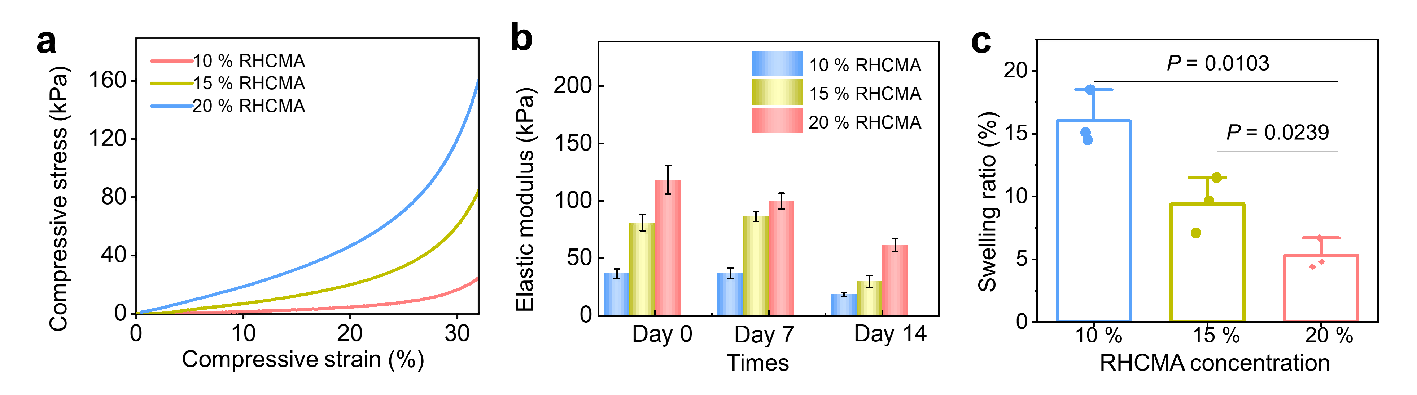


**Figure S6**. **Mechanical and swelling properties of RHCMA hydrogels.** a) Compression stress-strain curves for hydrogels derived from the mixture of RHCMA at respective concentrations and 1.6% PEO at volume ratio of 9:1. b) Elastic modulus for hydrogels in different groups at various time points (n=3). c) Swelling ratio of hydrogels in different groups (n=3). Data are shown as mean ± SD. n represents biological replicates.


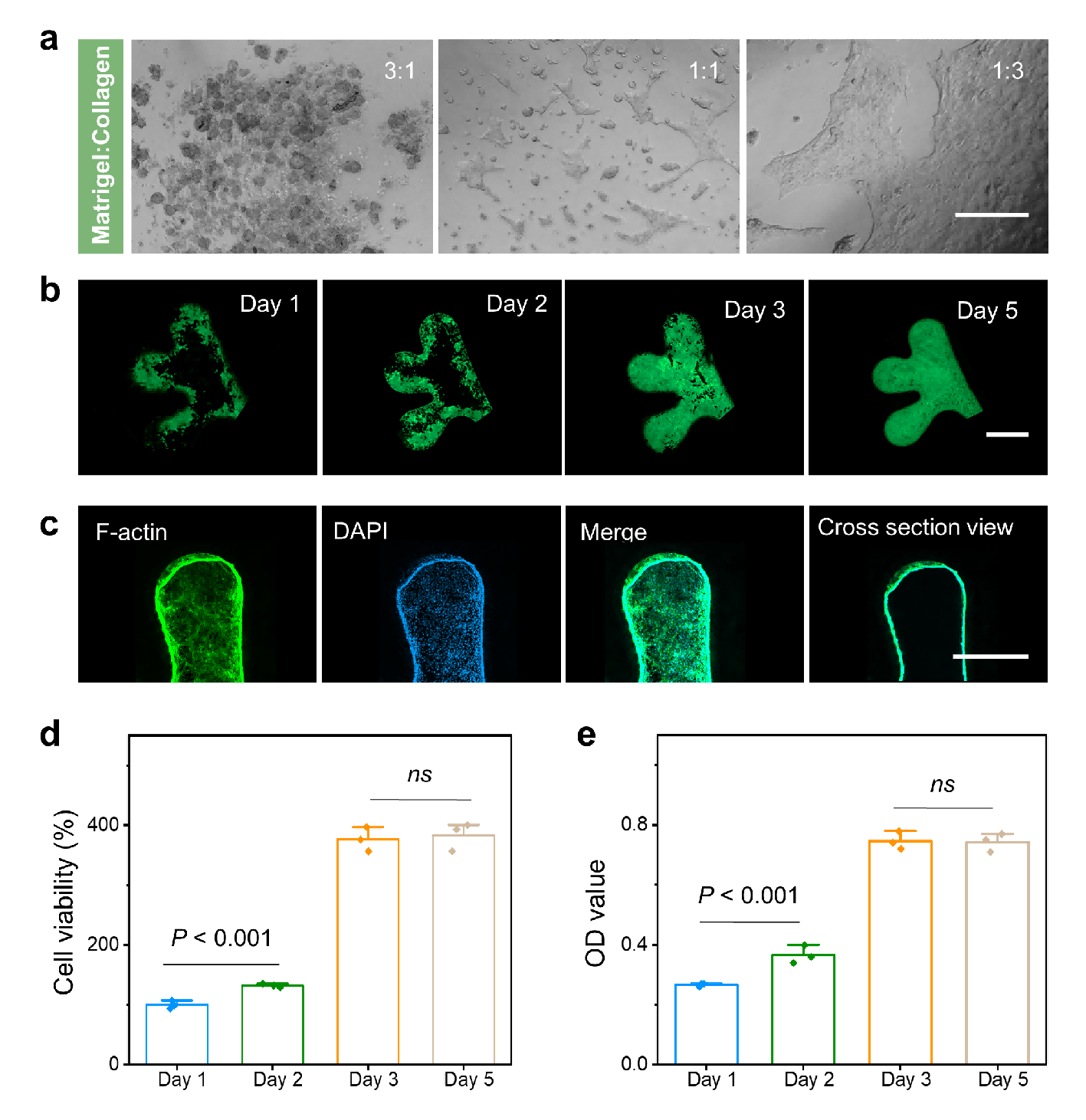


**Figure S7. Cell morphology and viability of MECs cultured on Col-Mat hydrogels and within the microfluidic chip.** a) Bright-field images showing the MECs seeded on the surface of thin Col-Mat gel layers with different ratios. b) Calcein AM staining of MECs cultured on-chip at different growth days. c) 3D layer-scanning reconstructed image of F-actin (representing the cytoskeleton, green) and DAPI Staining on Day 5. The right image is the cross-section view. d) Statistical analysis of Calcein AM staining for cell viability on different days (n=3). e) CCK-8 absorbance statistics for cells on-chip on different days (n=3). The scale bar is 200 μm in a), 2 mm in b) and c). Data are shown as mean ± SD. n represents biological replicates.


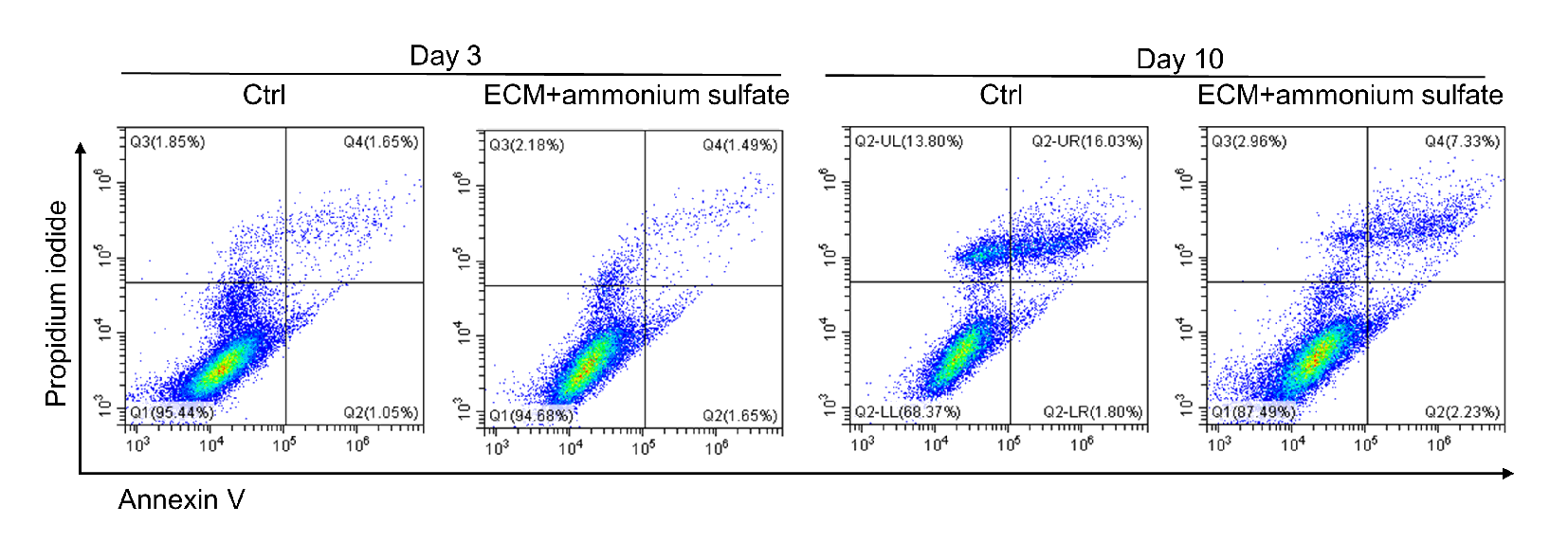


**Figure S8.** **Flow cytometry analysis of apoptosis under different treatments.**


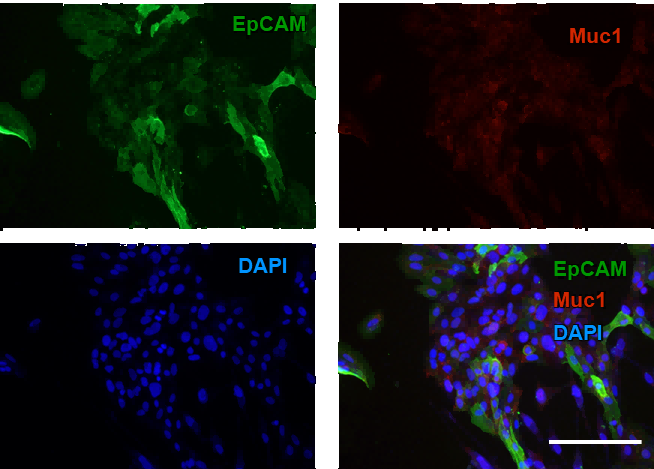


**Figure S9.** **Staining of stem/progenitor markers in MECs (MCF-10A) cultured on-chip one day after seeding.** The scale bar is 100 μm.


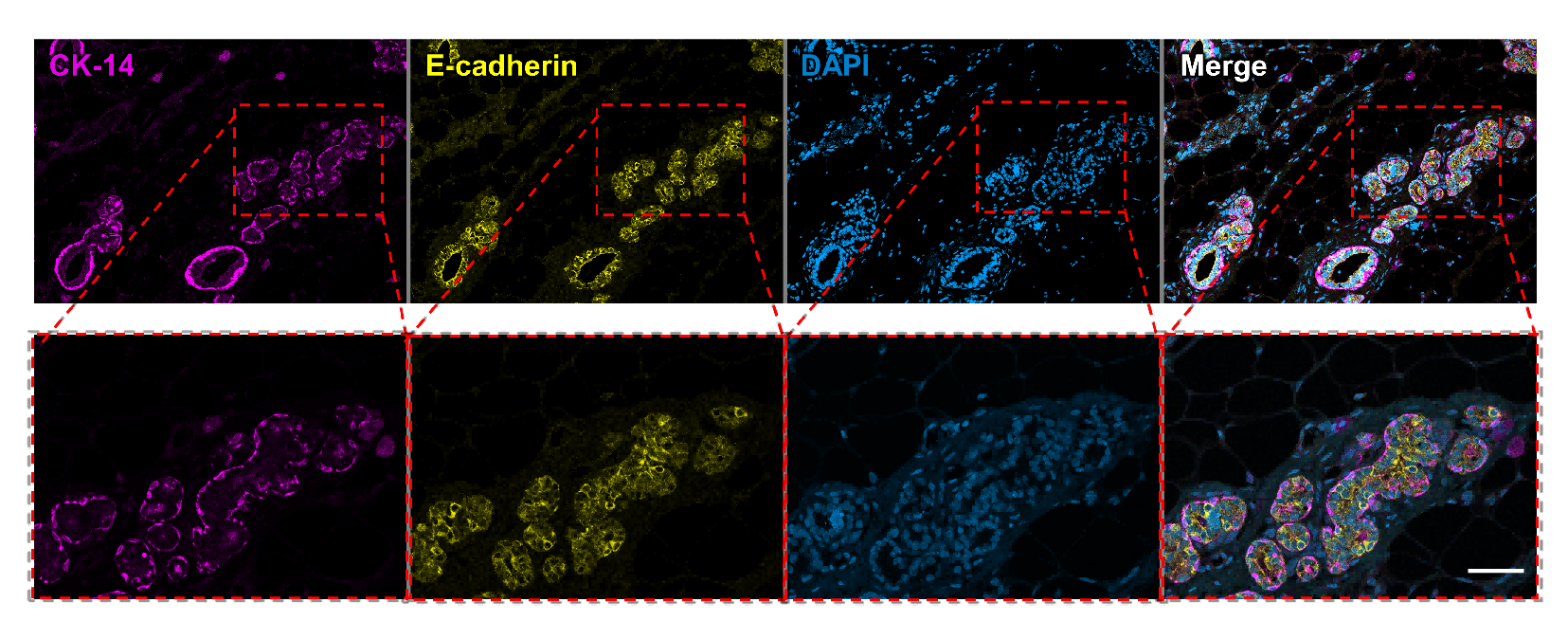


**Figure S10.** **Immunofluorescence staining of luminal (E-cadherin, yellow) and basal (CK14, purple) markers in rat mammary tissue.** The scale bar is 100 μm.


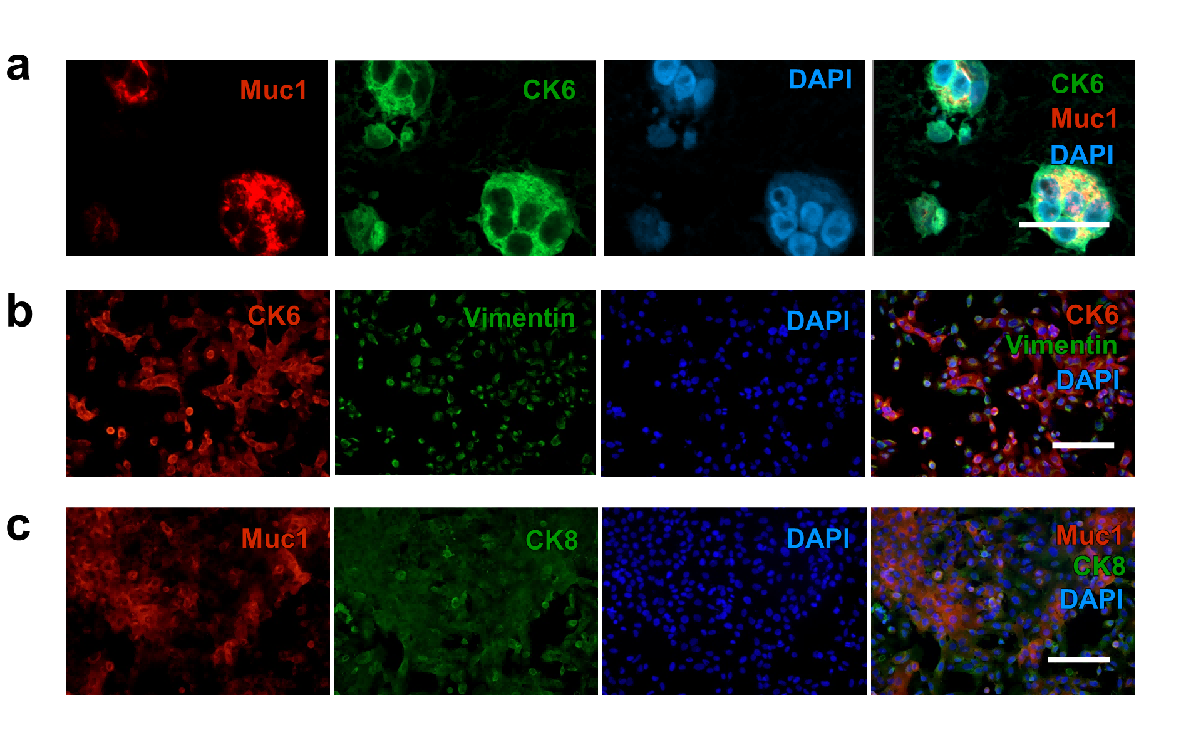


**Figure S11.** **Characterization of basal and luminal markers in MECs cultured within 3D Col-Mat hydrogel and on-chip.** a) Immunofluorescence staining of basal (CK6) and luminal (Muc1) markers for spheroids derived from MECs (MCF-10A) grown within a 3D Col-Mat hydrogel. b) Basal (CK6, Vimentin) and c) luminal (Muc1, CK8) epithelial staining of MECs on-chip after three days. The scale bar is 50 μm in a), and 100 μm in b-c).


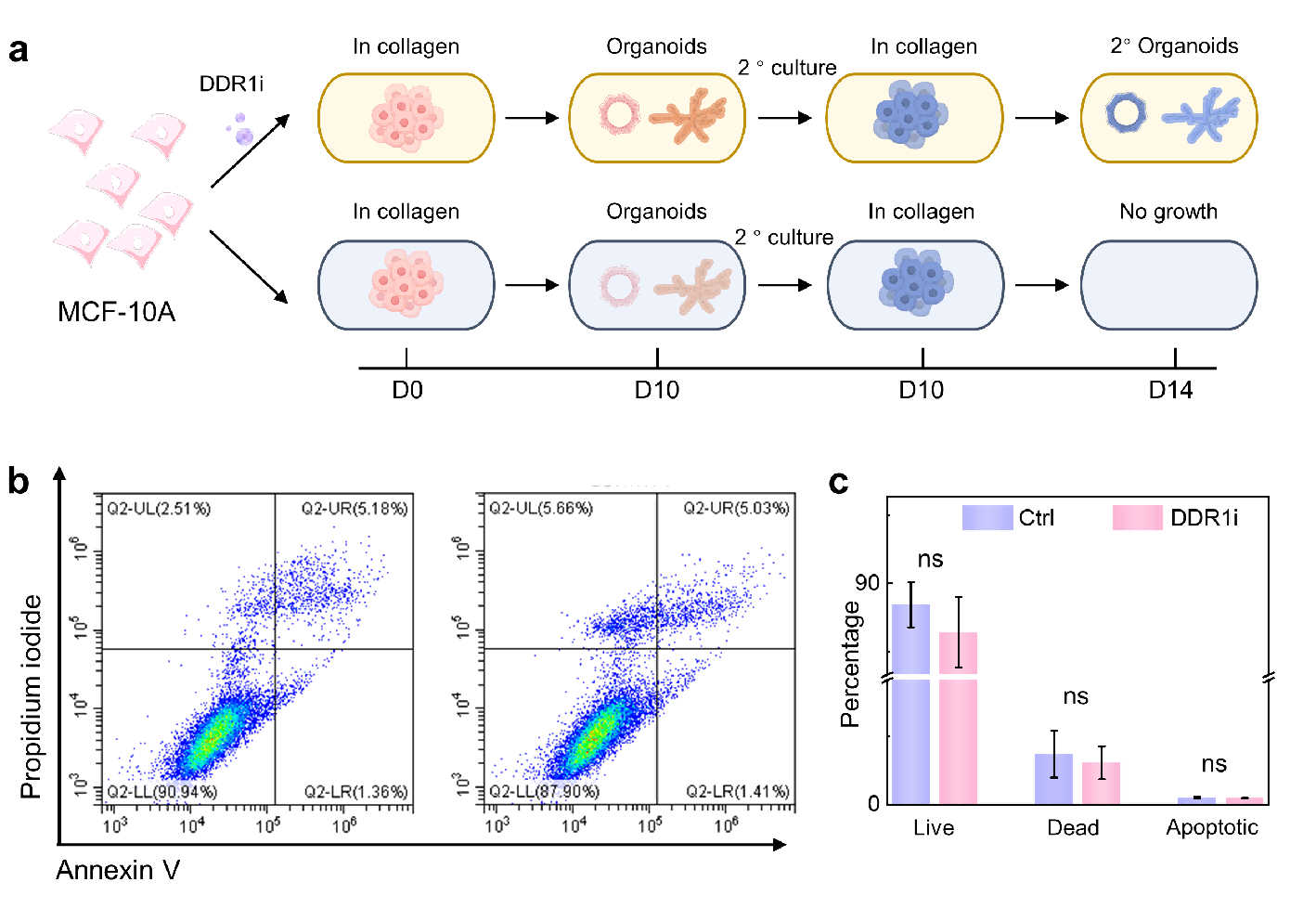


**Figure S12. Formation of primary and secondary mammary organoids in collagen hydrogel and apoptosis analysis.** a) Schematic diagram of primary (1°) organoid and secondary (2°) organoid formation when culturing in collagen hydrogel. b) Flow cytometry analysis of apoptosis. c) Statistics of Annexin V apoptosis results (n=3). Data are shown as mean ± SD. n represents biological replicates.


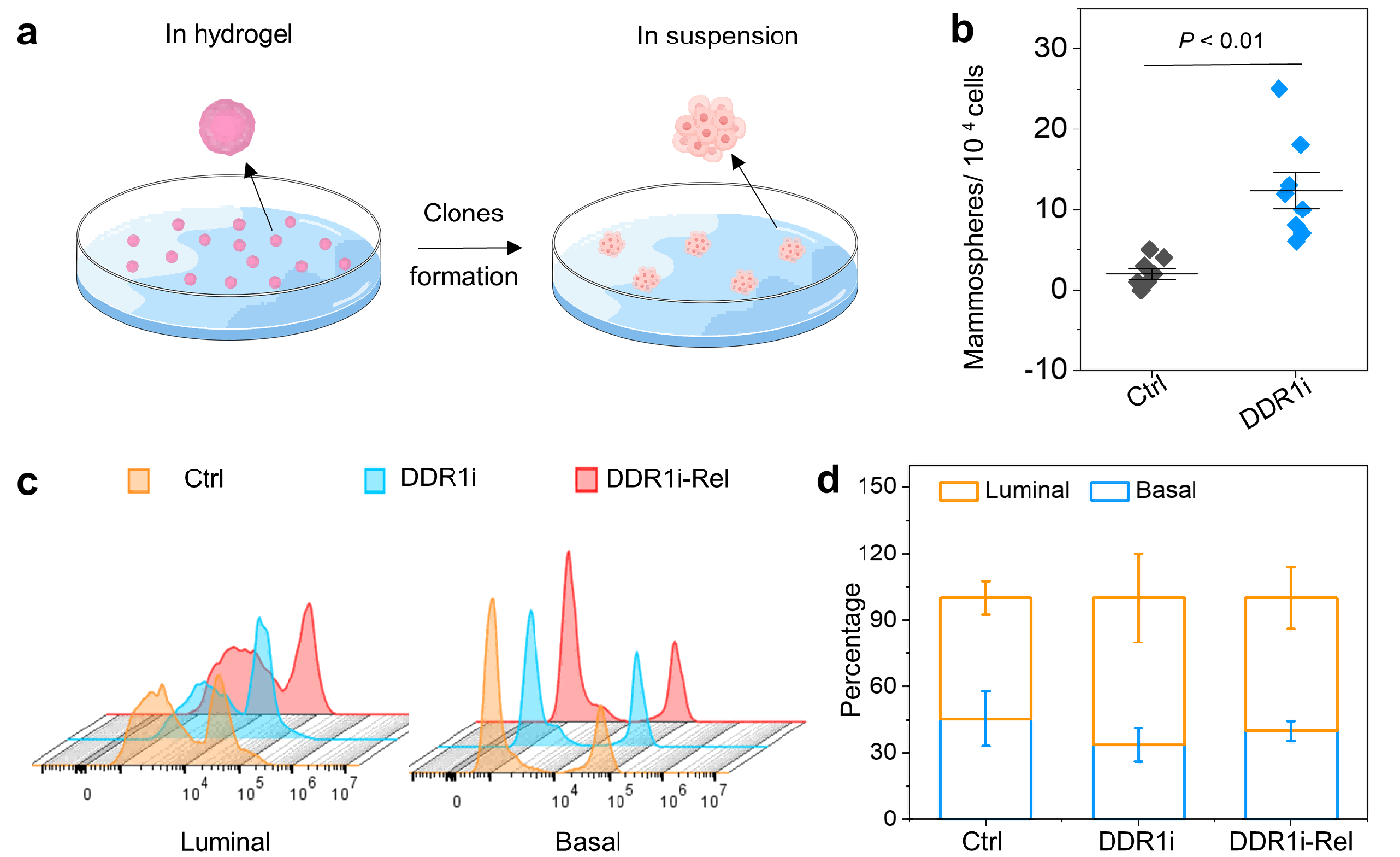


**Figure S13. Mammosphere formation assay and flow cytometry analysis of epithelial markers in primary mammary organoid cells.** a) Schematic of the mammosphere formation assay. b) Statistics of mammosphere formation in each group. c) Luminal (EpCAM) and basal (CD49f) expression in hydrogel-cultured primary mammary organoid cells across different groups. (d) Corresponding statistical analysis.


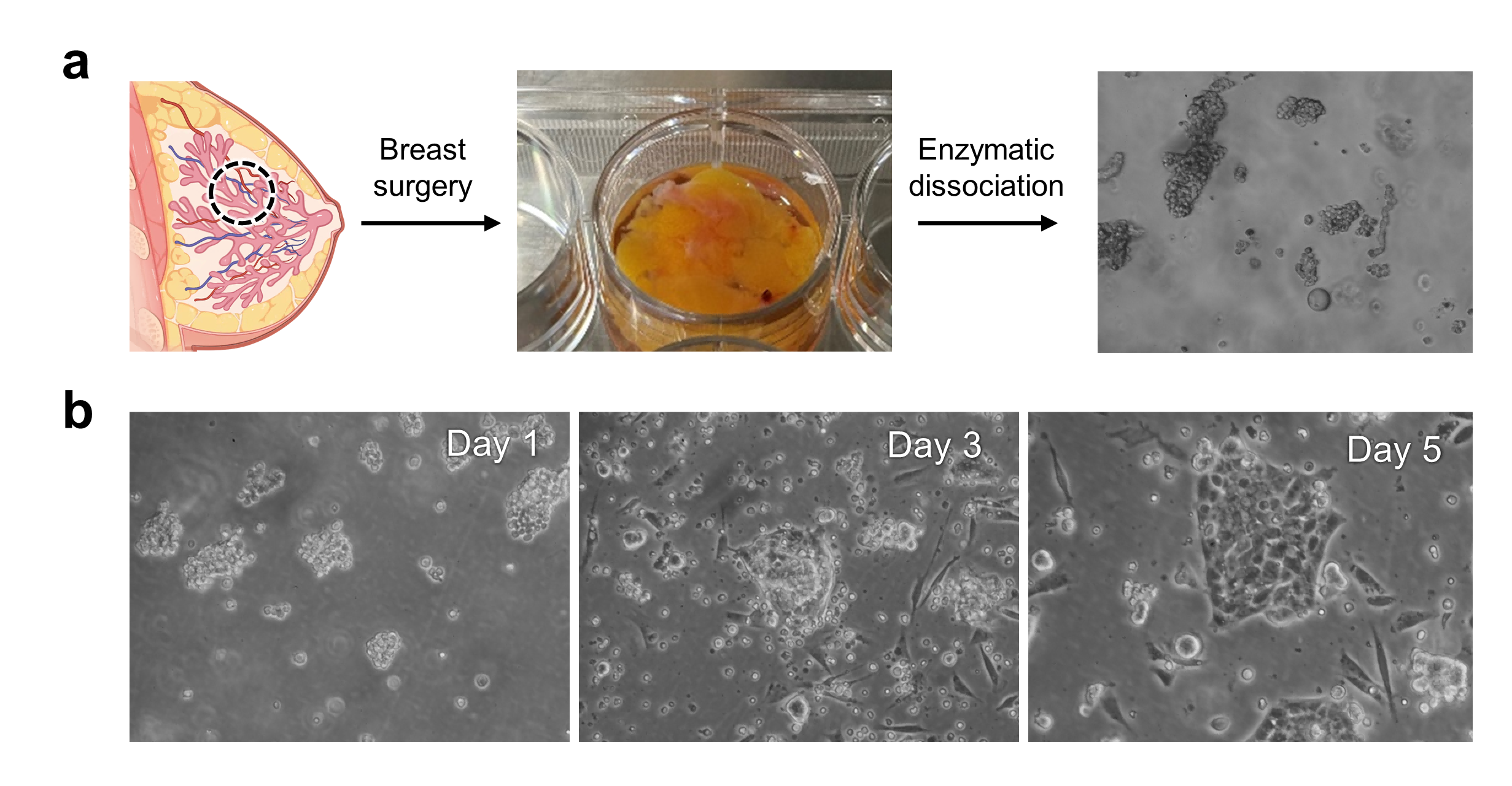


**Figure S14. Isolation and culture of primary MECs.** a) Primary MECs isolated from breast tissues. b) Bright-field images of MECs grown in different days.


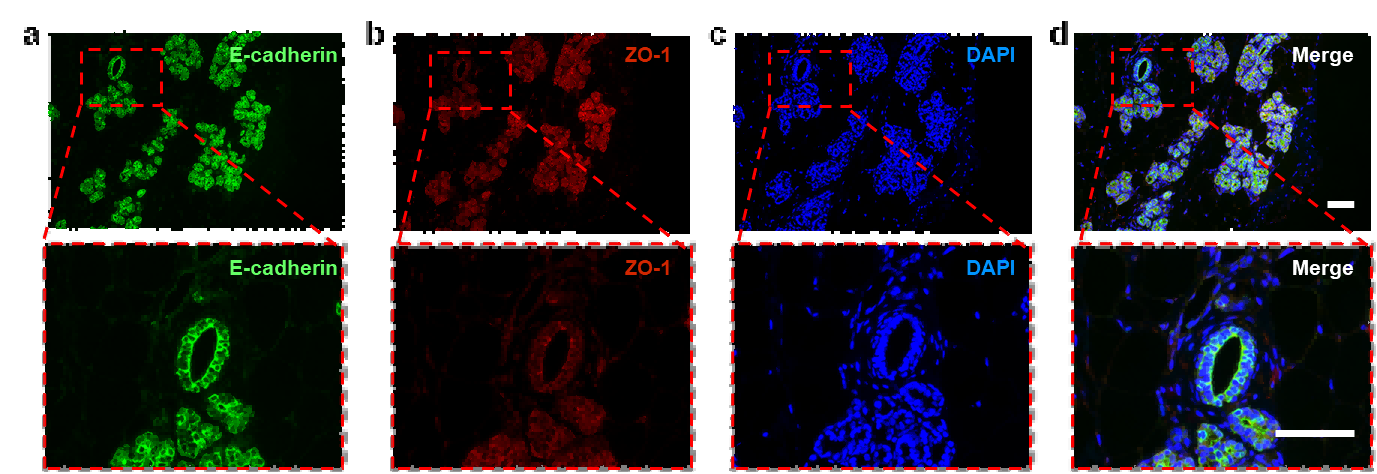


**Figure S15. Immunofluorescence staining of epithelial markers in rat mammary tissue.** Immunofluorescence staining of a) E-cadherin (green), b) TJ protein (ZO-1, red) and c) DAPI in rat mammary tissue. d) Merge image. The scale bar is 100 μm in d).


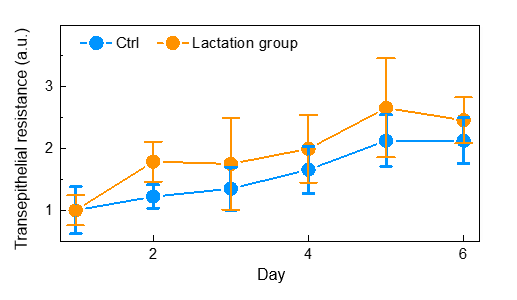


**Figure S16. Relative changes in transepithelial resistance under treatment of different culture medium** (n=5)**.** The control group was treated with growth medium and the lactation group received treatment with the lactation medium. Data are shown as mean ± SD. n represents biological replicates.


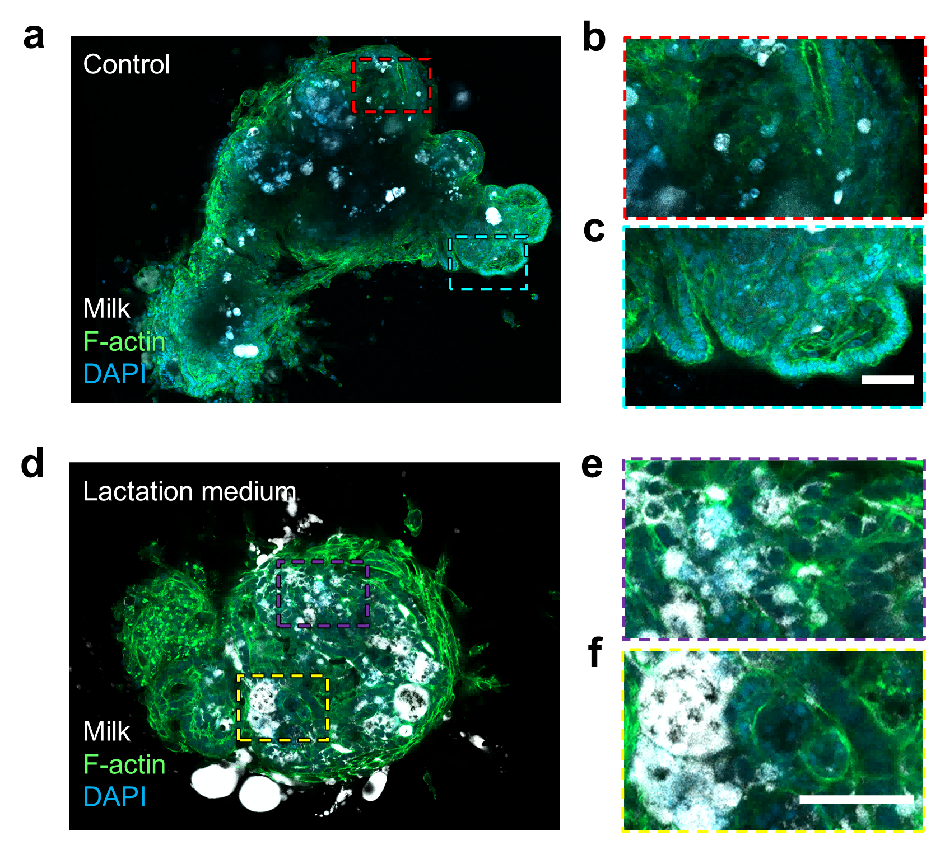


**Figure S17.** **Lipid droplet and F-actin staining in primary mammary organoids.** Nile red staining (lipid droplets, white) and F-actin staining (cytoskeleton, green) of primary mammary organoids grown within Matrigel a-c) before and d-f) after lactation medium stimulation. The scale bar is 50 μm in c) and f).


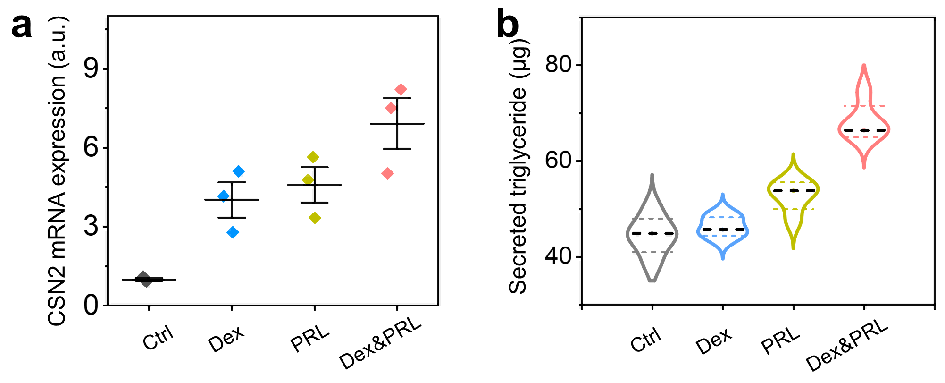


**Figure S18. Analysis of CSN2 mRNA expression and triglyceride secretion on-chip.** a) Relative mRNA expression of CSN2 (n=3). b) Statistics of total secreted triglyceride in different groups after ten days of culture, including three days of growth culture followed by seven days of lactation culture (n=3). Data are shown as mean ± SD. n represents biological replicates.


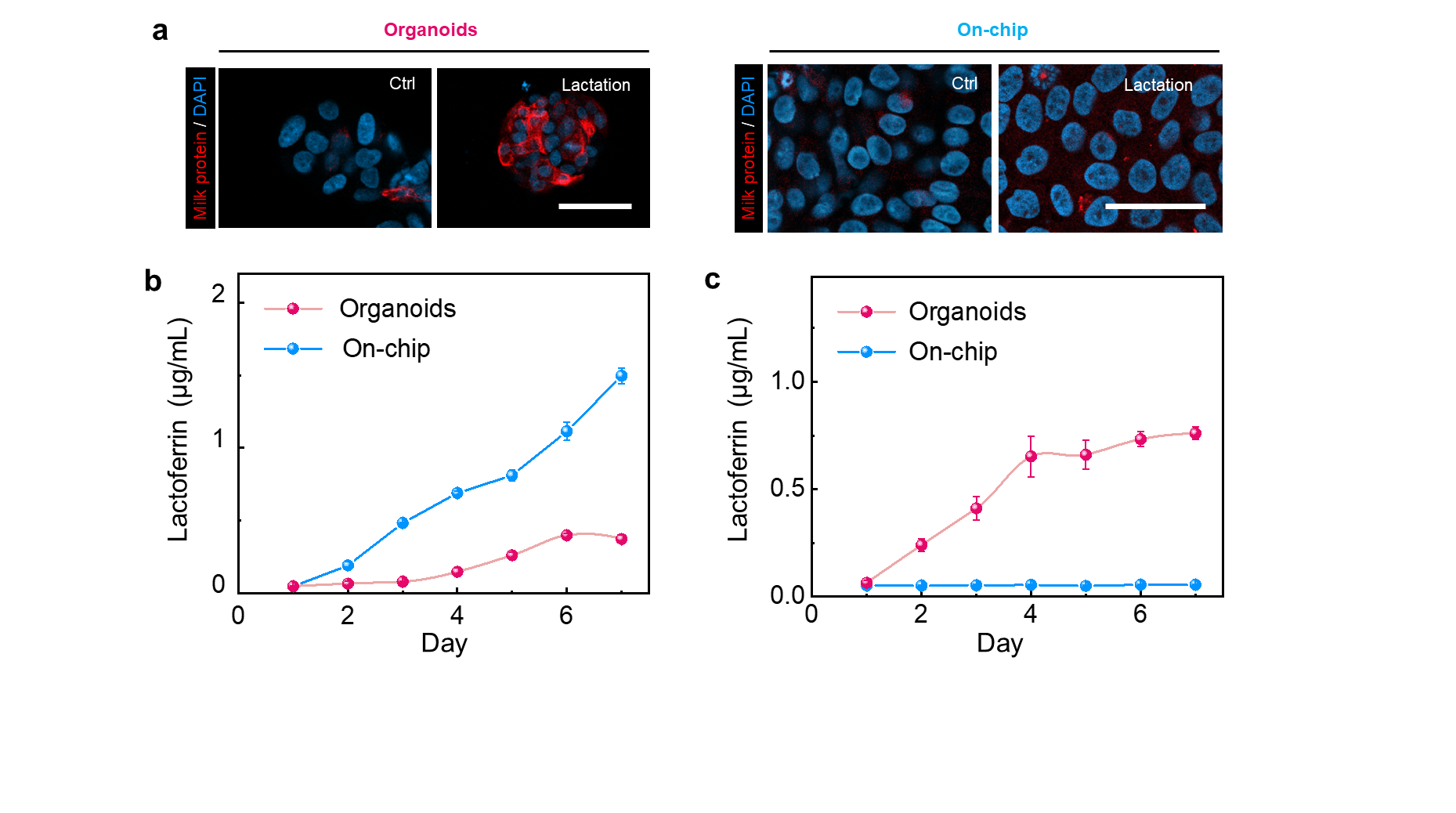


**Figure S19.** **Lactoferrin expression in primary organoids and MECs on-chip.** a) Immunofluorescence staining of lactoferrin in primary organoids within Matrigel and primary MECs on-chip. The control group was supplied with growth medium for six days, while the lactation group was provided with growth medium for the first three days followed by lactation medium for the subsequent three days. b) Quantification of lactoferrin content in the medium collected from the chamber in different groups ( n=5). c) Quantification of intracellular lactoferrin content in different groups. The scale bar is 50 μm in a). Data are shown as mean ± SD. n represents biological replicates.


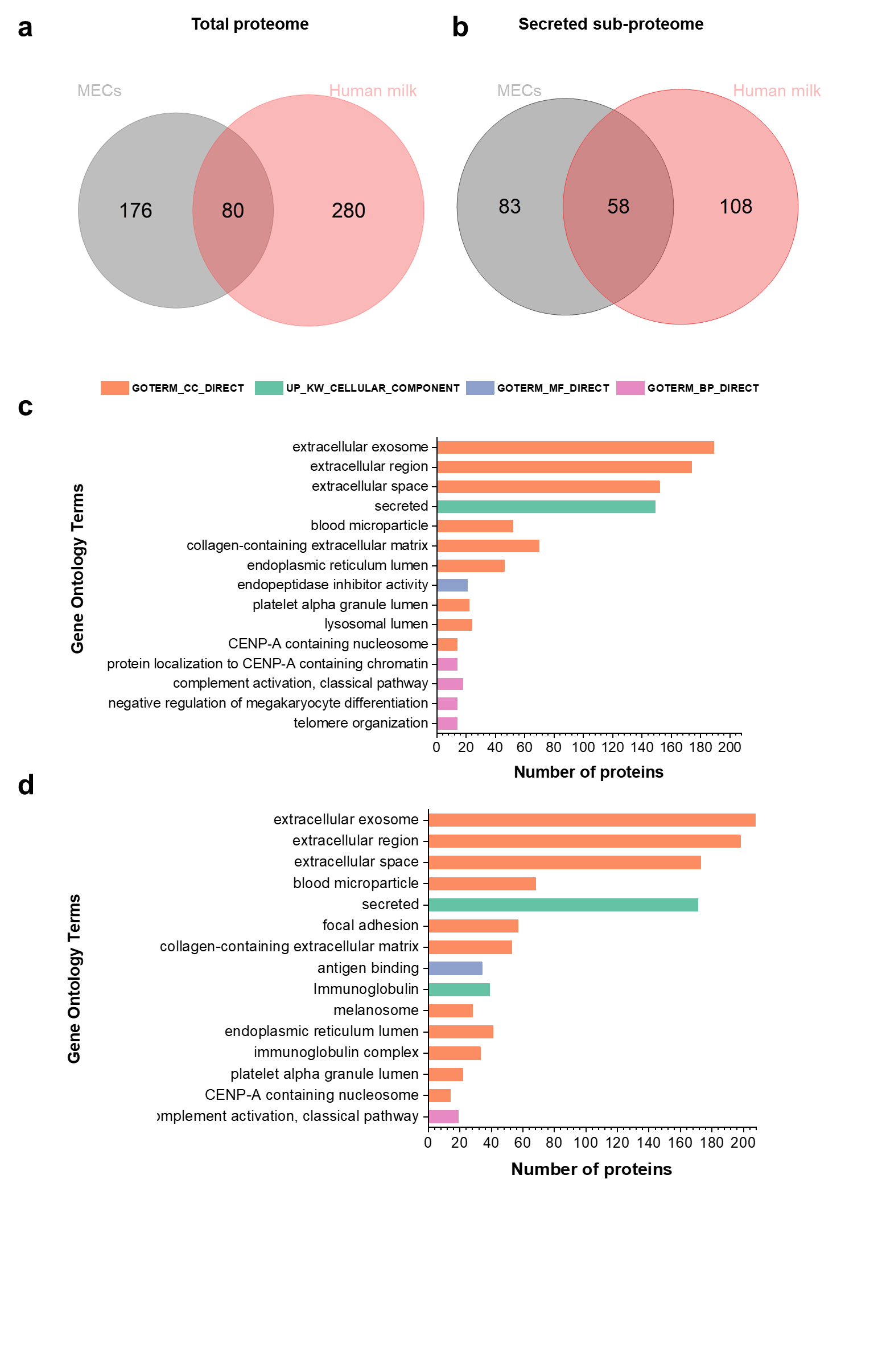


**Figure S20. Proteomic comparison between human milk and MECs secretome.** a) Venn diagram showing the overlap of total proteins identified in the MECs secretome and human milk. b) Venn diagram displaying the overlap within the secreted sub-proteome in different groups. c) Gene Ontology (GO) enrichment analysis for total proteome of MECs. d) Gene Ontology (GO) enrichment analysis for total proteome of human milk.


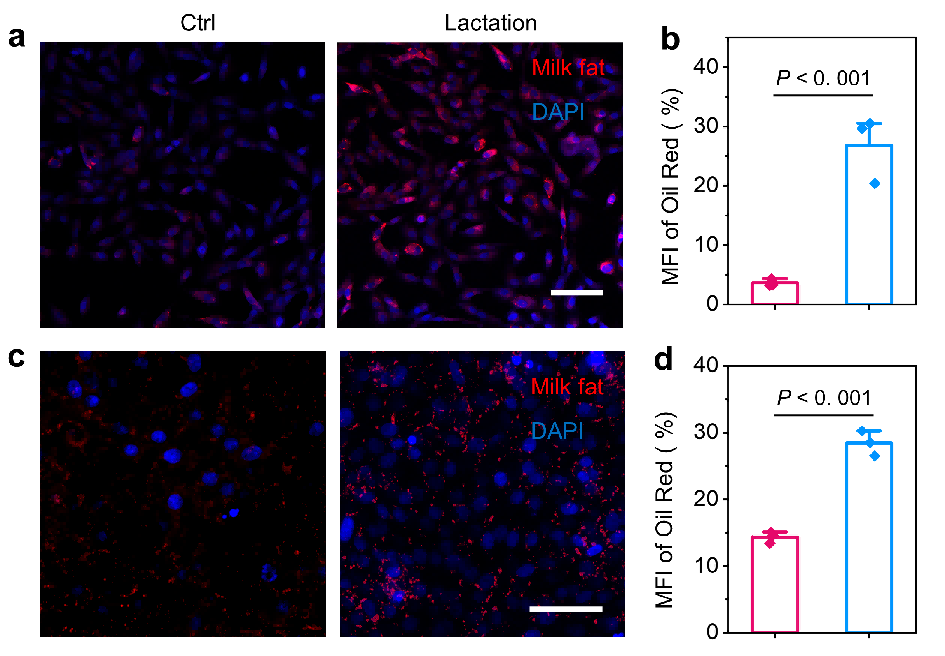


**Figure S21.** **Lipid droplet staining and fluorescence intensity analysis in different cell lines.** a) Lipid droplet staining of MCF-10A cells seeded on-chip. b) Corresponding relative fluorescence intensity. c) Lipid droplet staining of bovine MAC-T cells. d) Relative fluorescence intensity. The scale bar is 50 μm in c). Data are shown as mean ± SD. n represents biological replicates.


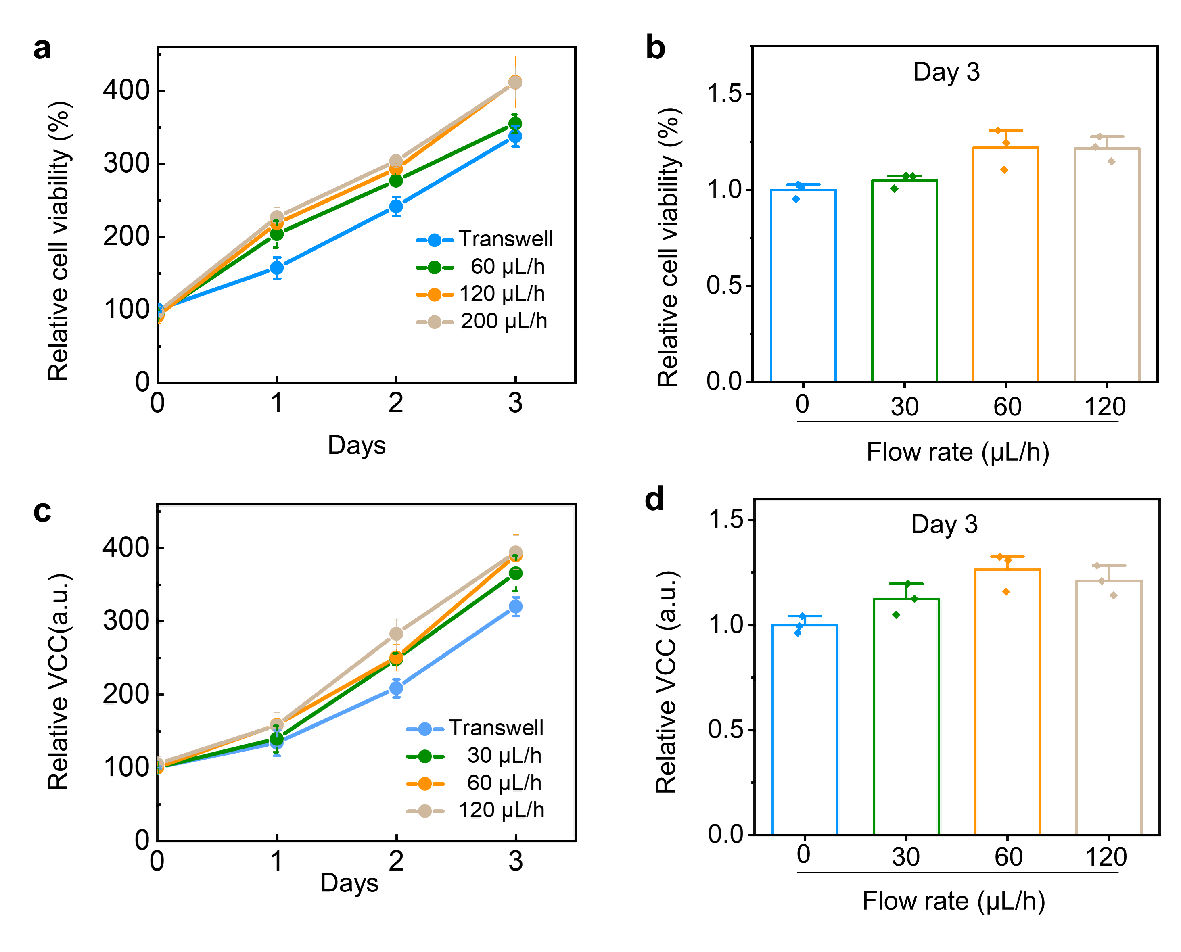


**Figure S22. Effect of flow rate on cell viability and viable cell count (VCC) during the growth phase.** a) Effect of flow rate on cell viability during the growth phase. The Transwell group corresponds to primary MECs cultured on Transwell systems with the same culture area. b) Relative cell viability on day 3. c) Effect of flow rate on VCC during the growth phase. d) Relative VCC on day 3. Data are shown as mean ± SD. n represents biological replicates.


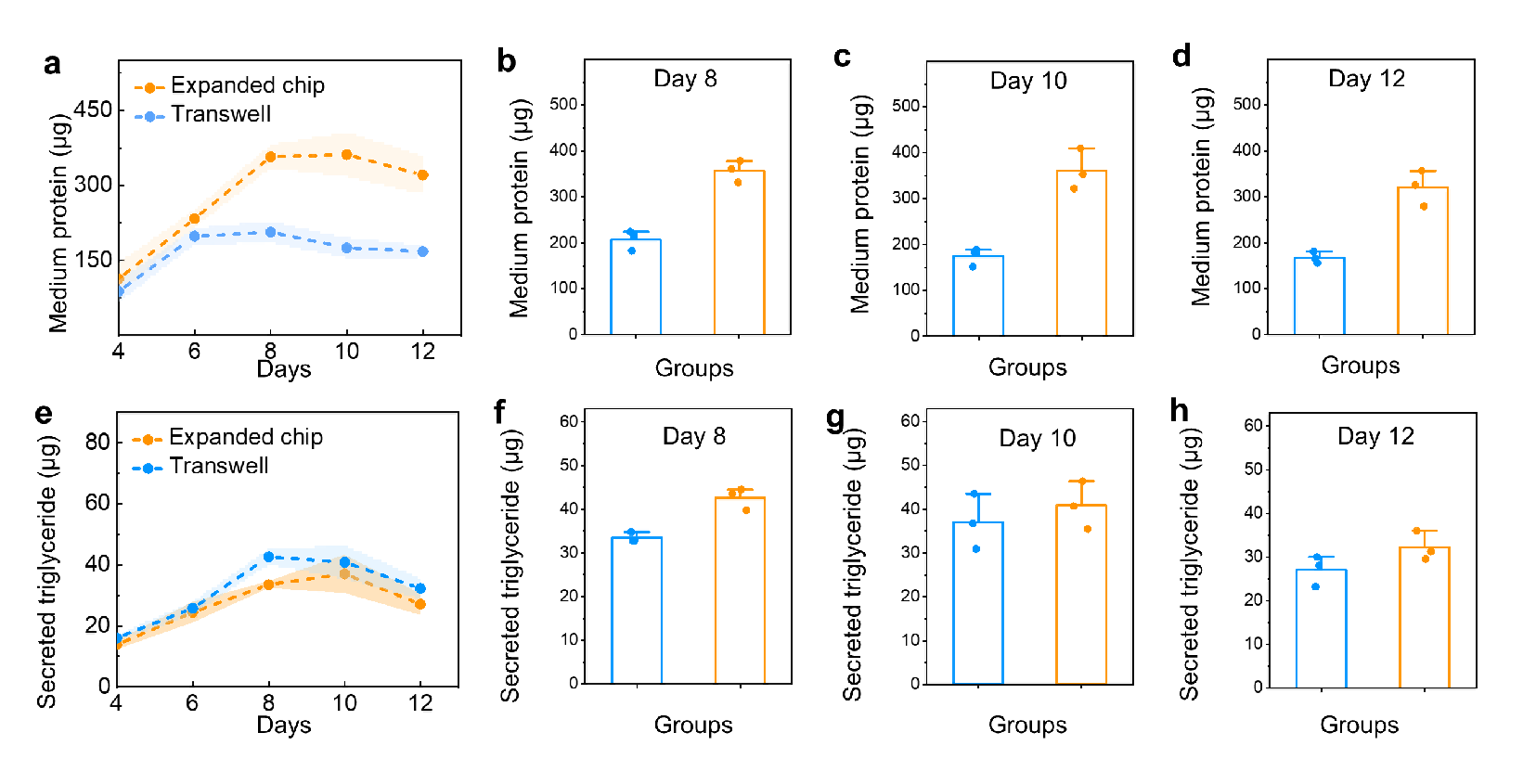


**Figure S23. Analysis of protein and triglyceride production during the lactation phase.** a) Statistics of total protein production of lactation medium during the lactation phase, b-d) Bar graphs representing data for different days. e) Statistics of triglyceride content in lactation medium during the lactation phase, f-h) Bar graphs representing data for different days. Data are shown as mean ± SD. n represents biological replicates.

**
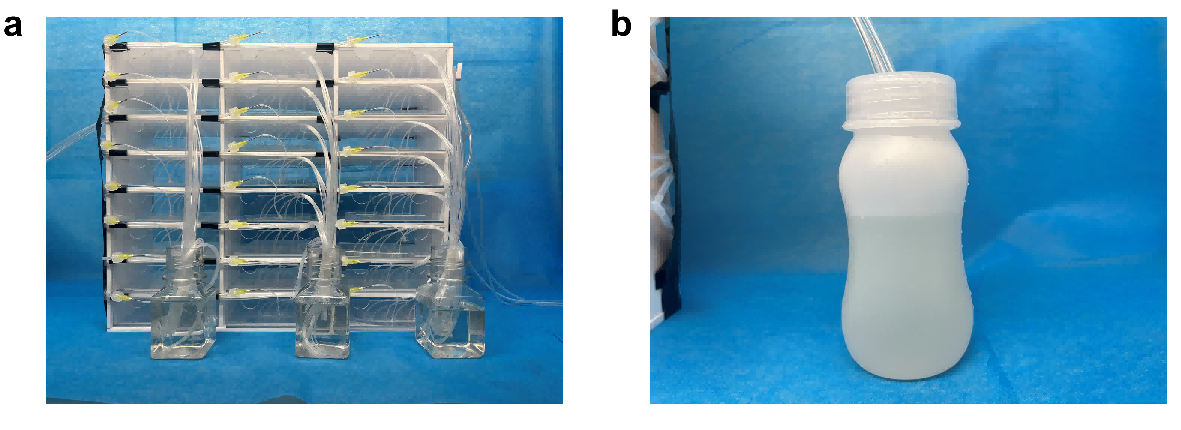
**

**Figure S24.** **Photos of Multilayer Chip Back and Collection Section.** a) Photograph of the back of multilayer chip. b) The collection part of the integrated mammary microphysiological system

**
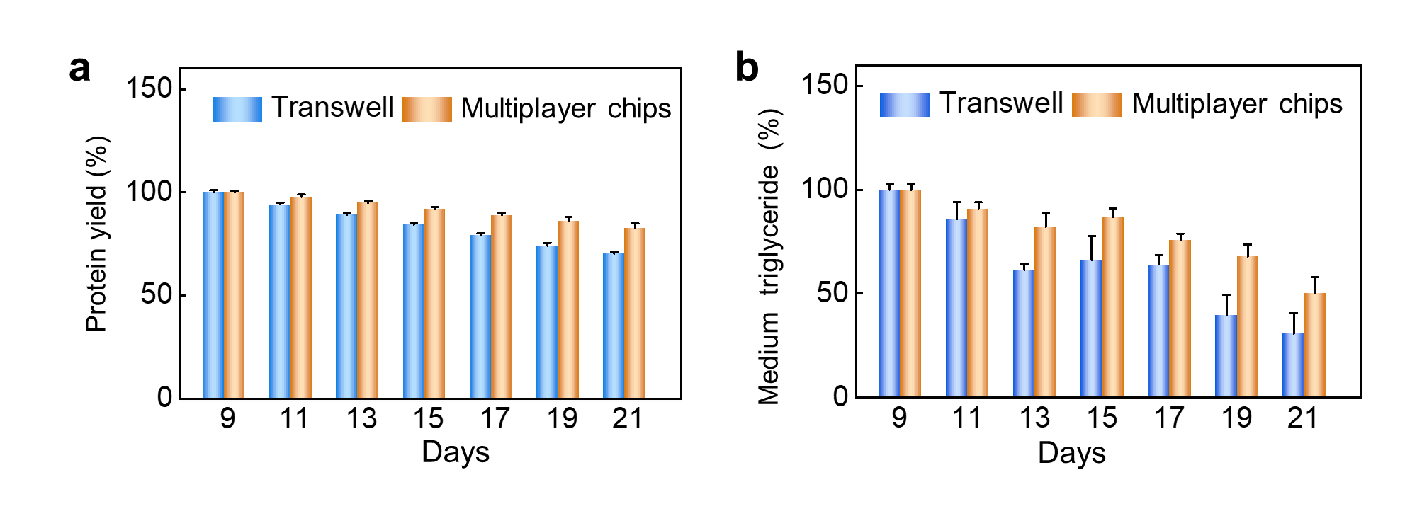
**

**Figure S25. Long-term culture analysis of protein and triglyceride yields.** a) Statistical analysis of total protein yield during long-term culture. b) Statistical analysis of triglyceride yield during long-term culture. Data are shown as mean ± SD. n represents biological replicates.

**Table S1.** Primers designed for mRNA detection

| **Gene name** | **Primer** |
| --- | --- |
| Human GAPDH F | TCCACTGGCGTCTTCACC |
| Human GAPDH R | GGCAGAGATGATGACCCTTTT |
| Human CSN2 F | TGCAAGGGAGACCATAGAAAGC |
| Human CSN2 R | CTTGCTGCTGGTCCTCATGTTT |
| Human LTF F | GTTTTGGCGCCTACTGAGGT |
| Human LTF R | ACTTTGGGGTACAAGCCACC |
| Human UGP2 F | GCAGGAGCAAAATGCCATTGACA |
| Human UGP2 R | CAGAAAACGGCTCCTTGGCACA |
| Human FABP3 F | TCATAACCTTCCCCCTACCCTC |
| Human FABP3 R | TCATCGAACTCCACCCCCAA |
| Human SREBP1 F | ACTTCTGGAGGCATCGCAAGCA |
| Human SREBP1 R | AGGTTCCAGAGGAGGCTACAAG |
